# Regulation of arginine transport by GCN2 eIF2 kinase is important for replication of the intracellular parasite *Toxoplasma gondii*

**DOI:** 10.1371/journal.ppat.1007746

**Published:** 2019-06-13

**Authors:** Leonardo Augusto, Parth H. Amin, Ronald C. Wek, William J. Sullivan

**Affiliations:** 1 Department of Biochemistry & Molecular Biology, Indiana University School of Medicine, Indianapolis, Indiana, United States of America; 2 Department of Pharmacology & Toxicology, Indiana University School of Medicine, Indianapolis, Indiana, United States of America; 3 Department of Microbiology & Immunology, Indiana University School of Medicine, Indianapolis, Indiana, United States of America; Johns Hopkins University Bloomberg School of Public Health, UNITED STATES

## Abstract

*Toxoplasma gondii* is a prevalent protozoan parasite that can infect any nucleated cell but cannot replicate outside of its host cell. *Toxoplasma* is auxotrophic for several nutrients including arginine, tryptophan, and purines, which it must acquire from its host cell. The demands of parasite replication rapidly deplete the host cell of these essential nutrients, yet *Toxoplasma* successfully manages to proliferate until it lyses the host cell. In eukaryotic cells, nutrient starvation can induce the integrated stress response (ISR) through phosphorylation of an essential translation factor eIF2. Phosphorylation of eIF2 lowers global protein synthesis coincident with preferential translation of gene transcripts involved in stress adaptation, such as that encoding the transcription factor ATF4 (CREB2), which activates genes that modulate amino acid metabolism and uptake. Here, we discovered that the ISR is induced in host cells infected with *Toxoplasma*. Our results show that as *Toxoplasma* depletes host cell arginine, the host cell phosphorylates eIF2 via protein kinase GCN2 (EIF2AK4), leading to induced ATF4. Increased ATF4 then enhances expression of the cationic amino acid transporter CAT1 (SLC7A1), resulting in increased uptake of arginine in *Toxoplasma*-infected cells. Deletion of host GCN2, or its downstream effectors ATF4 and CAT1, lowers arginine levels in the host, impairing proliferation of the parasite. Our findings establish that *Toxoplasma* usurps the host cell ISR to help secure nutrients that it needs for parasite replication.

## Author summary

Parasites that live inside a host cell must develop strategies to ensure sufficient delivery of nutrients required for survival and replication. After invasion, *Toxoplasma* rapidly usurps the supply of its essential amino acid arginine from the host cell. Sensing low levels of arginine, the host cell initiates a nutrient starvation response designated the integrated stress response (ISR) that leads to enhanced expression of CAT1, a transporter that facilitates arginine uptake. Through activation of the host ISR and increased expression of this transporter, *Toxoplasma* secures a continued supply of arginine for its growth and reproduction. Inhibition of these pathways by therapeutic intervention could be a novel strategy to impair survival of the intracellular parasite.

## Introduction

*Toxoplasma gondii* is an obligate intracellular protozoan parasite that can infect any nucleated cell. *Toxoplasma* resides and replicates within a non-fusogenic parasitophorous vacuole that functions to siphon nutrients from its host cell [1]. As an intracellular pathogen, *Toxoplasma* is auxotrophic for a range of nutrients, including tryptophan, arginine, polyamines, purines, and cholesterol, and relies on its host cell to supply them [2]. Parasites rendered incapable of salvaging these nutrients from host cells suffer reduced growth and virulence. For example, *Toxoplasma* lacking TgNPT1, a selective arginine transporter, show decreased survival [3]. A major unresolved question is how intracellular parasites, such as *Toxoplasma*, are able to ensure that a continued supply of essential nutrients is available as they rapidly replicate in host cells.

Phosphorylation of the α subunit of eukaryotic initiation factor-2 (eIF2α) is a well-characterized response to amino acid starvation. Mediated by the protein kinase GCN2 (EIF2AK4), phosphorylation of eIF2α (eIF2α-P) lowers translation initiation, which serves to conserve nutrients and energy [4]. Accompanying repression in global protein synthesis, eIF2α-P also enhances the translation of select mRNAs involved in stress adaptation. An example of a preferentially translated gene target is *ATF4*, which encodes a transcription factor that directs amino acid metabolism and transport, antioxidation, and cell survival [5]. In addition to GCN2, there are three other mammalian eIF2α kinases: PERK (EIF2AK3/PEK), PKR (EIF2AK2) and HRI (EIF2AK1), which are activated by endoplasmic reticulum (ER) stress, viral infection, and heme deprivation in reticulocyte cells, respectively [6]. Because eIF2α-P can induce *ATF4* translation in response to different stresses, this pathway is referred to as the integrated stress response (ISR) [7].

This study addresses the mechanisms by which *Toxoplasma* ensures that its host cell continues to provide sufficient nutrients for parasite replication. We show that upon *Toxoplasma* infection, host cells become depleted for amino acids such as arginine, a nutrient stress that triggers the host ISR. Specifically, *Toxoplasma* infection prompts GCN2 phosphorylation of eIF2α in host cells, which leads to increased expression of *ATF4*. Enhanced levels of ATF4 triggered transcriptional expression of *CAT1* (*SLC7A1*), which encodes a cationic transporter that facilitates arginine uptake by the host cell, thereby maintaining a ready supply for rapidly replicating parasites. Deletion of any component of the host GCN2/ATF4/CAT1 pathway lowers arginine levels in *Toxoplasma*-infected cells, dramatically reducing parasite replication.

## Results

### *Toxoplasma* infection activates the ISR through GCN2

**Fig 1:**
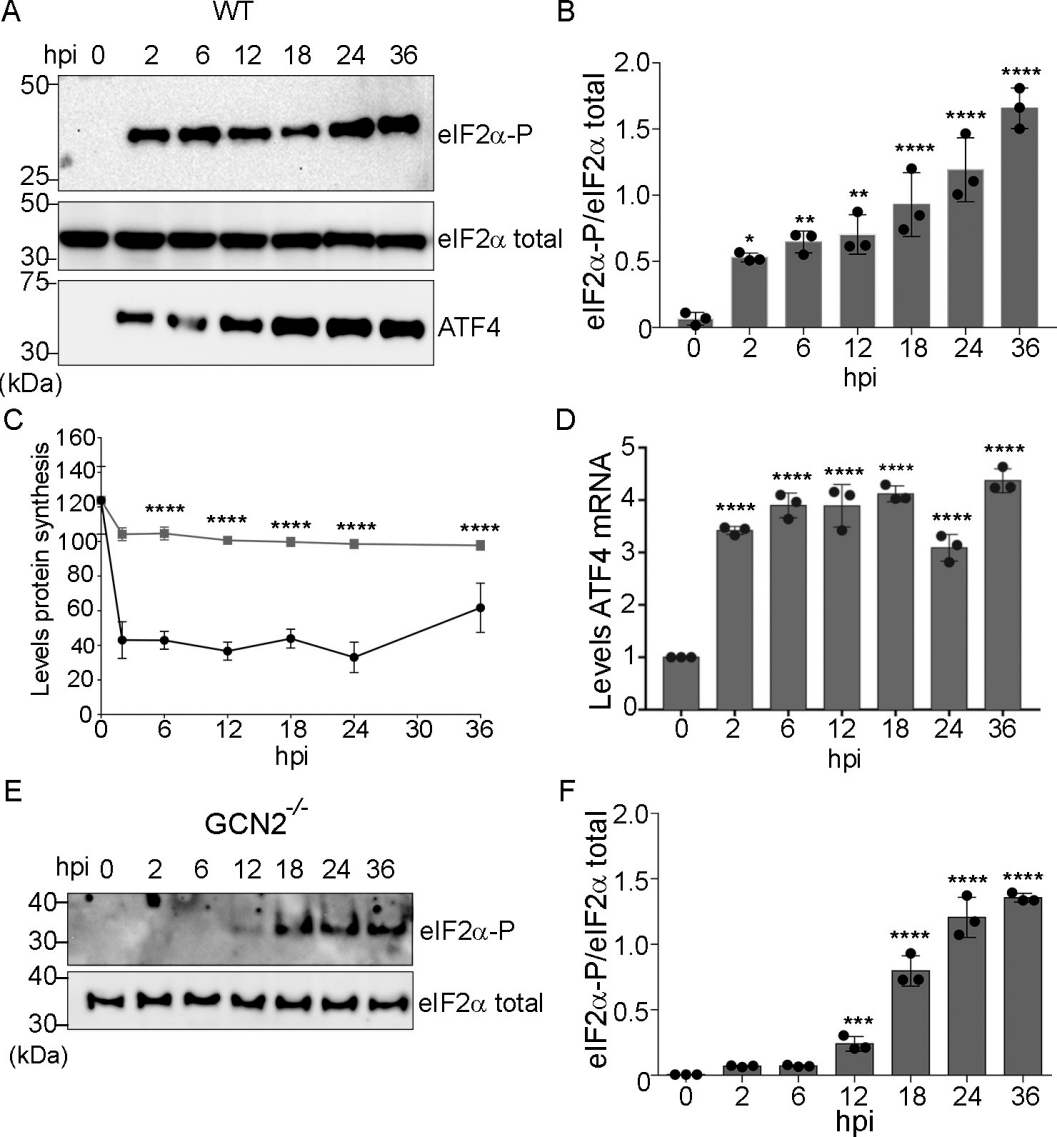
*Toxoplasma* infection triggers activation of GCN2 and induced eIF2α phosphorylation in host cells. **(A)** Wild-type (WT) MEF cells were infected with *Toxoplasma* using a MOI of 3:1. At the indicated hpi, infected cells were harvested and the levels of eIF2α-P, total eIF2α, and ATF4 were measured by immunoblot. **(B)** Bar graph showing the levels of eIF2α-P normalized for total eIF2α at each of the infection time points. Amounts of eIF2α-P were normalized to total eIF2α (±SD, n = 3) *p<0.01, ** p<0.005, ****p<0.0001. **(C)** Total protein synthesis was measured in MEF cells infected with *Toxoplasma* (black, circles) or mock-infected (grey, squares) for the indicated time. Global translation was measured by incubating cells with puromycin for 15 min, followed by lysate preparation and immunoblot with puromycin-specific antibodies. The y-axis is densitometry analysis of the protein as measured by immunoblot normalized to total eIF2. (±SD, n = 3) ****p<0.0001. **(D)** *ATF4* mRNA levels were measured by RT-qPCR; values were normalized to mock-infected cells (±SD, n = 3) ****p<0.0001. **(E)** *GCN2*^-/-^ MEF cells were infected for the indicated time and levels of eIF2α-P and total eIF2α were measured by immunoblot. **(F)** Amounts of eIF2α-P were normalized to total eIF2α (±SD, n = 3), *** p<0.0005, ****p<0.0001.

We hypothesized that depletion of nutrients in *Toxoplasma*-infected cells would initiate the host ISR. To test this idea, we infected mouse embryonic fibroblast (MEF) cells with *Toxoplasma* and measured the level of eIF2α-P. Two hours after infection, MEF cells showed increased levels of eIF2α-P accompanied by a reduction in global protein synthesis **(Fig 1A–1C and S1A and S1B Fig)**. Coincident with increased ATF4 protein **(Fig 1A)**, infected host cells also showed increased *ATF4* mRNA levels **(Fig 1D),** both hallmark features of the ISR [4]. Induction of eIF2α-P was also observed upon infection of HFF cells, HEK293T cells, and J774.1 macrophages, albeit HFF cells showed some differences in the timing of induction **(S2A–S2E Fig)**. These findings indicate that the ISR can be activated in different types of host cells in response to *Toxoplasma* infection.

To determine if GCN2 activates the ISR during *Toxoplasma* infection, we infected MEF cells lacking GCN2 [8]. Following infection of *GCN2*^*-/-*^ cells, there was a significant delay in the induction of host eIF2α-P, with appreciable levels detected only after 18 hours post-infection (hpi) (**Fig 1E and 1F**) that was accompanied by a delay in the induction of *ATF4* mRNA levels **(S3A Fig)**. These data show that GCN2 is a “first responder” eIF2α kinase during *Toxoplasma* infection of host cells, but other eIF2α kinase(s) can function later during the course of infection.

**Fig 2:**
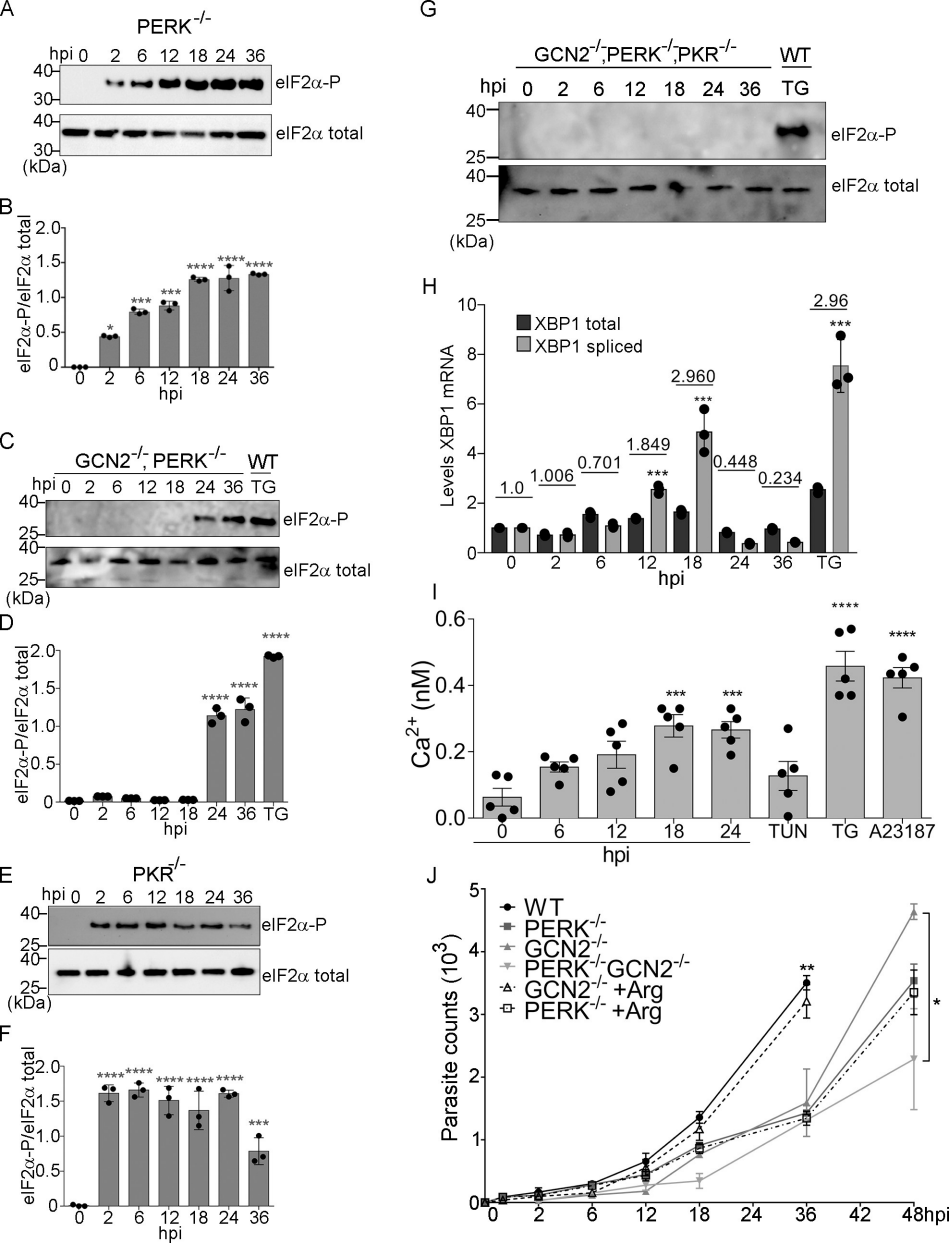
*Toxoplasma* infection causes ER stress. **(A)** *PERK*^-/-^ MEF cells were infected with *Toxoplasma* for the indicated times and the levels of eIF2α-P and total eIF2α were measured by immunoblot. **(B)** The ratio of eIF2α-P versus total eIF2α is represented in the bar graph (±SD, n = 3) *p<0.01, ***p<0.0005, ****p<0.0001. **(C-G)** MEF cells lacking the indicated eIF2α kinase(s) were infected with *Toxoplasma* for the designated times and the amounts of eIF2α-P and total eIF2α were measured by immunoblot. These experiments were repeated three times with similar results and one representative blot is shown. Thapsigargin (TG) was used as a positive control to induce ER stress and the ratio of eIF2α-P versus total eIF2α is represented in the bar graph (±SD, n = 3) ***p<0.0005, ****p<0.0001. **(H)** Levels of total and spliced *XBP1* mRNA were measured by RT-qPCR in MEF cells infected with *Toxoplasma* for the indicated hpi. Values are normalized to mock-infected cells for each time point. As a positive control, the ER stress agent thapsigargin (TG) was added to the MEF cells for 6 h (±SD, n = 3) ***p<0.0005, ****p<0.0001. Values indicate the ratio of total/spliced *XBP1* mRNA. **(I)** Ca^2+^ levels were measured by a colorimetric assay in uninfected WT MEF cells and those infected with *Toxoplasma* for the indicated times. As expected, uninfected cells treated with the calcium ionophore A21387 or the SERCA inhibitor thapsigargin (TG) showed high levels of cytosolic calcium, whereas treatment with the ER stress agent tunicamycin (TUN), which inhibits N-glycosylation, showed minimal change in calcium levels. Values of infected cells were normalized to mock-infected cells (±SD, n = 3), ***p<0.005, ****p<0.0001. **(J)** Wild-type (WT) MEF cells and those deleted for *GCN2* and *PERK* individually or in combination, as indicated, were infected with *Toxoplasma*. Infected MEF cells lacking GCN2 or PERK were supplemented with arginine (100-fold of DMEM medium) (dashed lines). At the indicated times, genomic DNA was extracted and qPCR used to quantify the number of parasites in the host cells. Data were analyzed with multiple t-test (±SD, n = 3), *p<0.01 and ***p<0.001.

In the ISR, a primary eIF2α kinase is activated in response to a given stress, with one or more secondary eIF2α kinases being induced with extended cell perturbations [9]. To identify the host eIF2α kinase(s) that serve as secondary ISR responders during *Toxoplasma* infection, we infected MEF cells lacking PERK or PKR, or multiple eIF2α kinases [10]. While *PERK*^*-/-*^ cells showed robust eIF2α-P early in infection along with a rise in *ATF4* mRNA levels starting at 2 hpi **(Fig 2A and 2B, S3B Fig)**, eIF2α-P was detected in the combined *GCN2*^*-/-*^ *PERK*^*-/-*^ cells only after 24 hpi, followed by an increase in *ATF4* mRNA levels at 36 hpi **(Fig 2C and 2D, S3C Fig**). In cells lacking *PKR*, there was no detectable change in eIF2α-P or *ATF4* mRNA levels during *Toxoplasma* infection until 36 hpi **(Fig 2E and 2F, S3D Fig)**. MEF cells lacking *GCN2*, *PERK*, and *PKR* completely ablated induction of the host ISR during *Toxoplasma* infection, with no measureable eIF2α-P and minimal *ATF4* mRNA even after 36 hpi **(Fig 2G, S3E Fig)**. These findings suggest that PKR may perform a modest role in the induction of the ISR late during infection (after 24 hpi). The lack of host eIF2α-P in the triple knock out MEF cells also suggests that the eIF2α kinase HRI does not play a significant role throughout infection **(Fig 2G)**. Our results suggest that host GCN2 is activated early in *Toxoplasma* infection, with induction of the secondary eIF2α kinase PERK (which is activated by ER stress) occurring later in the course of infection. Consistent with the idea that *Toxoplasma* infection produces ER stress in the host cell, we found that activation of IRE1, an ER-resident riboendonuclease that facilitates cytosolic splicing of *XBP1* mRNA [11], occurs 12 hpi **(Fig 2H)**. Furthermore, there were increased levels of cytosolic calcium in infected cells **(Fig 2I, S4C and S4D Fig)**, a feature reported to occur upon disruption of the ER [12].

Our measurements of eIF2α-P in infected MEF cells suggest that *Toxoplasma* initially activates GCN2, followed by PERK at ~18 hpi. The inability of host cells to induce the ISR has a detrimental effect on *Toxoplasma* infection. Parasite replication was decreased nearly 50% in MEF cells lacking either GCN2 or PERK or both at 36 hpi **(Fig 2J)**. Interestingly, at 48 hpi, parasites grew more slowly in MEF cells lacking both GCN2 and PERK compared to MEF cells lacking either GCN2 or PERK, suggesting that optimal parasite growth relies on both of these host eIF2 kinases (**Fig 2J**). Supplementation with arginine rescues parasite replication in MEF cells lacking GCN2 but not in MEFs lacking PERK **(Fig 2J)**. These findings show that the host ISR is a significant contributor to robust *Toxoplasma* replication.

### Activation of GCN2 occurs via arginine depletion during *Toxoplasma* infection

**Fig 3:**
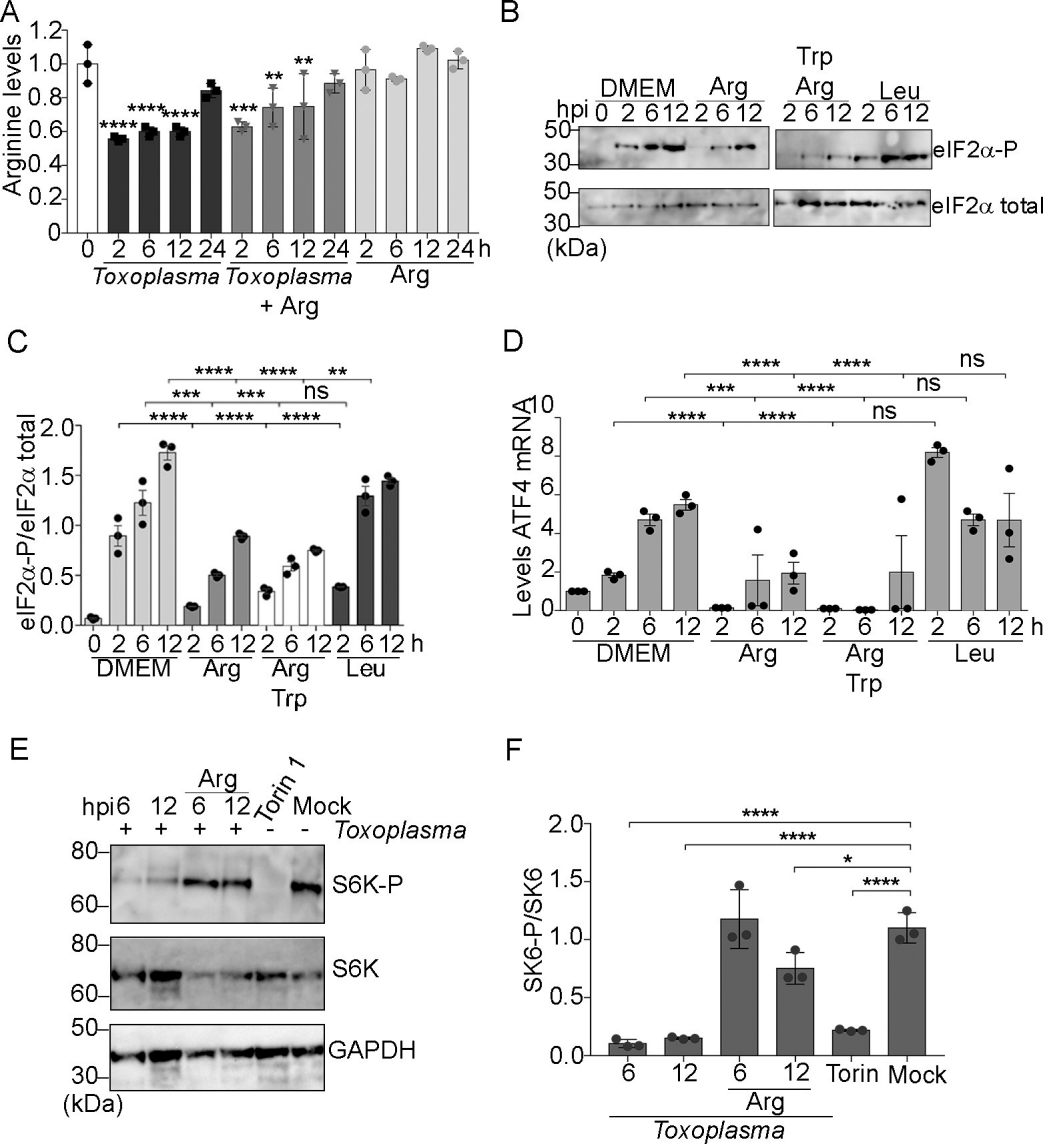
Arginine depletion caused by *Toxoplasma* infection induces eIF2α-P. **(A)** Levels of arginine were measured in uninfected and infected cells in the absence or presence of supplemented arginine. Uninfected cells are indicated as 0 h, which was normalized to 1 in the histogram. Values of infected cells were normalized to mock-infected cells (±SD, n = 3) **p<0.001, ****p<0.0001. **(B-C)** Cells infected with *Toxoplasma* were supplemented with 100-fold arginine, tryptophan, or leucine present in DMEM, and the levels of eIF2α-P and eIF2α-total were measured by immunoblot. The bar graph represents the ratio of eIF2α-P normalized for total eIF2α (±SD, n = 3) ***p<0.0005 and ****p<0.0001. **(D)** *ATF4* mRNA levels were measured by RT-qPCR in *Toxoplasma*-infected MEF cells supplemented with the indicated amino acids. Levels of *ATF4* mRNA were normalized to mock-infected cells (±SD, n = 3) **p<0.001, ***p<0.0005, ****p<0.0001. **(E)** Host cells infected with *Toxoplasma* were cultured in presence or absence of arginine, as indicated. As controls, cells were treated with mTORC1 inhibitor (Torin 1, for 1 h) or mock-infected. As expected, Torin 1 ablated S6K phosphorylation. Equal amounts of protein lysates were analyzed by immunoblot to determine the levels of total S6K, S6K-P, and GAPDH. **(F)** The bar graph represents the amount of S6K-P normalized for total S6K shown in (E) (±SD, n = 3) *p<0.05 and ****p<0.0001.

We next tested whether depletion of an essential amino acid, such as arginine, occurs during *Toxoplasma* infection, contributing to activation of host GCN2. Coincident with the time during infection when GCN2 is activated (2–12 hpi), host arginine levels were depleted; arginine was reduced by more than 40% within 2 hpi and remained low 12 hpi **(Fig 3A)**. Of note, a partial restoration in arginine levels was observed 24 hpi **(Fig 3A)**.

To further test the influence of arginine depletion during *Toxoplasma* infection on the activation of host GCN2, we supplemented the culture medium with additional amounts of arginine, tryptophan, or leucine (*Toxoplasma* is auxotrophic for arginine and tryptophan). The addition of 100-fold arginine to the medium significantly delayed and lowered levels of host eIF2α-P and *ATF4* mRNA during infection **(Fig 3B–3D);** similar results were obtained with just a 10-fold supplementation of arginine to the DMEM medium **(S5 Fig)**. By comparison, supplementation with leucine did not alleviate the ISR in infected host cells. The combined addition of arginine and tryptophan further lowered eIF2α-P during infection compared to arginine alone, suggesting that host tryptophan availability may be also affected during infection **(Fig 3B–3D)**. Furthermore, supplementation with arginine can rescue parasite replication in MEF cells deleted for *GCN2* (Fig 2J). These results bolster the model that activation of GCN2 during *Toxoplasma* infection occurs as a consequence of lowered availability of amino acids in the host cells, with arginine being a predominant nutrient required for *Toxoplasma* replication.

Another host protein kinase regulated by arginine depletion is mTORC1, which regulates many cellular processes including protein synthesis [13]. As amino acid starvation represses mTORC1, there is decreased phosphorylation of its substrate, S6 kinase (S6K). Upon *Toxoplasma* infection and the accompanying depletion of host cell arginine, we found that S6K phosphorylation was rapidly reduced in the host; supplementing the infected cells with arginine partially restored the phosphorylation of the mTORC1 substrate **(Fig 3E and 3F)**.

### Host arginine transporter CAT1 facilitates *Toxoplasma* replication

**Fig 4:**
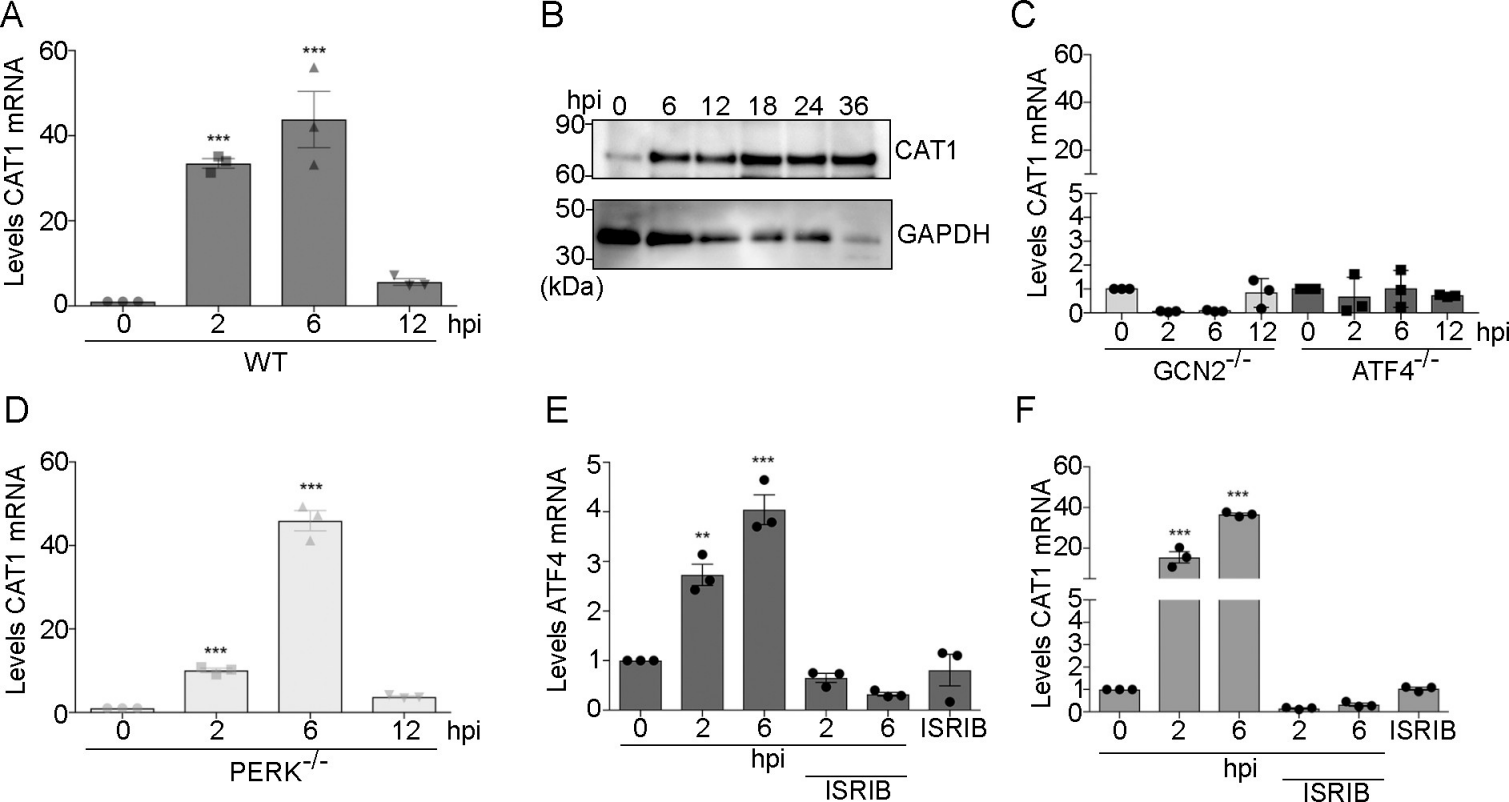
Host GCN2/ATF4 pathway induces *CAT1* expression upon *Toxoplasma* infection. **(A)** Levels of *CAT1* mRNA were measured by RT-pPCR in wild-type (WT) MEF cells infected with *Toxoplasma* for up to 12 h. Values of infected cells were normalized to mock-infection. Error bars represent standard deviation (n = 3) ***p<0.0005, ****p<0.0001. **(B)** Levels of CAT1 protein were measured by immunoblot in WT MEF cells infected with *Toxoplasma* for up to 36 h. As a normalization control, GAPDH protein levels were measured in the same lysate preparation. N = 3; numbers below the immunoblot indicate the densitometry values for CAT1/GAPDH. **(C)** *GCN2*^-/-^ and *ATF4*^-/-^ or **(D)** *PERK*^-/-^ MEF cells were infected with *Toxoplasma* for the indicated time. Levels of *CAT* mRNA were measured by RT-qPCR, and the values of infected cells were normalized to mock-infected cells. Error bars show standard deviation (n = 3) ***p<0.0005, ****p<0.0001. **(E-F)** WT MEF cells were infected for the indicated times in presence or absence of the ISR inhibitor ISRIB. Levels of *ATF4* and *CAT1* mRNAs were measured by RT-qPCR. Values of infected cells were normalized to mock-infected cells (±SD, n = 3) ***p<0.0005.

We sought to further elucidate the mechanism by which the host ISR is co-opted to ensure that sufficient levels of arginine are available for *Toxoplasma* replication. Transcriptional and translational expression of the arginine transporter CAT1 (*SLC7A1*) is induced by amino acid depletion [14,15]. We found increased levels of *CAT1* mRNA in infected host cells at 2 and 6 hpi, which were partially diminished by 12 hpi **(Fig 4A)**. Furthermore, we detected elevated levels of CAT1 protein at 6 hpi that were sustained throughout the time course of *Toxoplasma* infection **(Fig 4B and S6A Fig)**. By comparison, mRNA expression of the related cationic amino acid transporter genes, *SLC7A2* and *SLC7A3*, did not change after infection, suggesting that these transporters do not play a major role during *Toxoplasma* infection **(S7A and S7B Fig)**.

Deletion of *GCN2* or its downstream effector *ATF4* in MEF cells significantly lowered the induction of *CAT1* mRNA upon infection with *Toxoplasma*, with no change in protein levels **(Fig 4C, S6B and S6C Fig)**. By contrast, the absence of PERK, which does not respond directly to amino acid depletion and is activated later in infection, did not diminish the induction of *CAT1* transcript at 6 hrs **(Fig 4D, S6D Fig)**. Treatment of infected cells with ISRIB, a small molecule that blocks eIF2α-P induction of the ISR [16], lowered the induced expression of both *ATF4* and *CAT1* **(Fig 4E and 4F, S6E Fig).** These results indicate that GCN2-mediated phosphorylation of eIF2α, and the ensuing induction of ATF4, enhances *CAT1* expression in response to *Toxoplasma* infection.

### *Toxoplasma* replication is impaired in CAT1-depleted host cells

**Fig 5:**
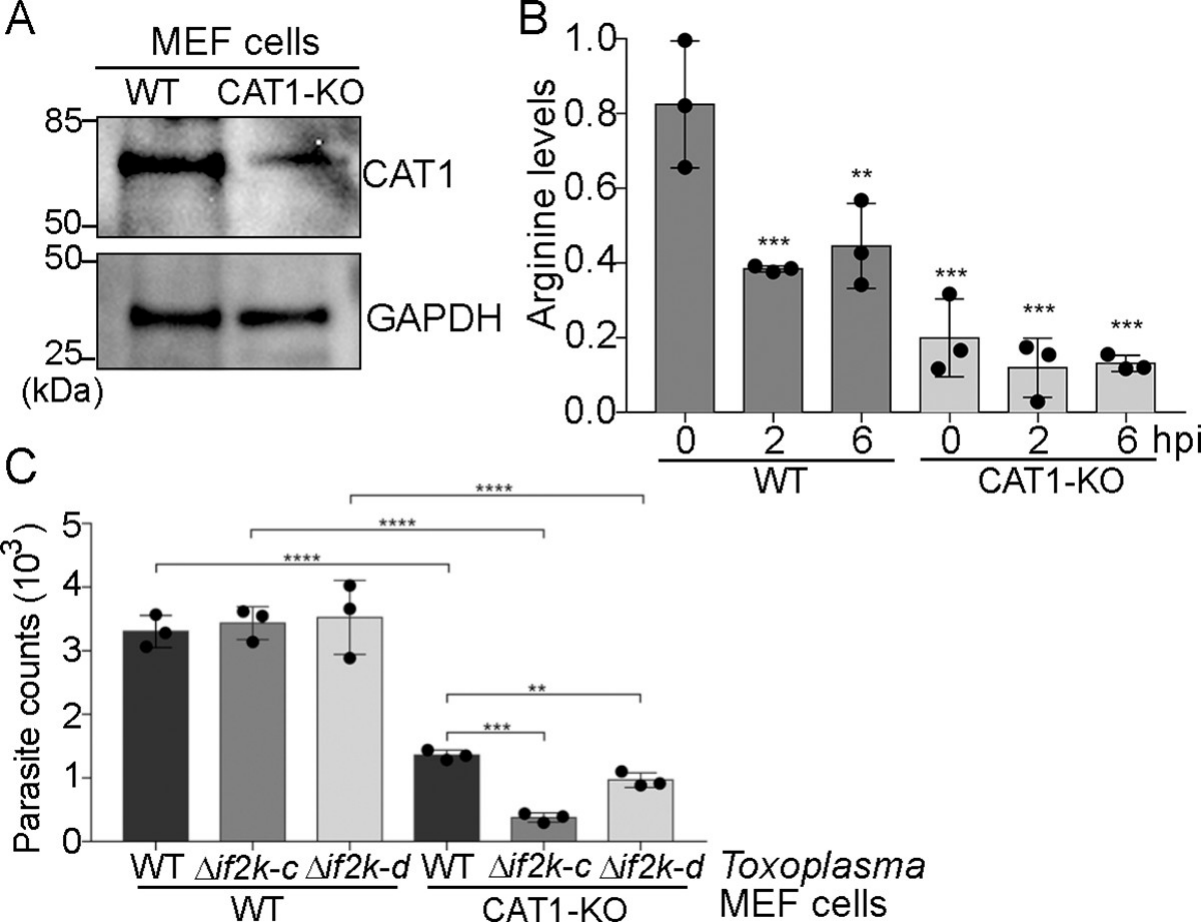
CAT1 facilitates replication of *Toxoplasma*. **(A)** *CAT1* was depleted from MEF cells using CRISPR-Cas9 and levels of CAT1 protein were measured by immunoblot. **(B)** Levels of host arginine were measured in wild-type (WT) MEF cells or those depleted for *CAT1* (CAT1-KO) and infected with *Toxoplasma* for 2 or 6 h. Values of infected cells were normalized to mock-infected cells, indicated as 0 hpi (±SD, n = 3) **p<0.001, ***p<0.0005. **(C)** At 30 hpi, genomic DNA was isolated and numbers of parasites inside WT or CAT1-KO MEF cells were determined by a qPCR assay. WT *Toxoplasma* parasites or those deleted for TgIF2K-C or TgIF2K-D were used to infect the indicated MEF cells for 30 hpi. Error bars represent standard deviation (n = 3). **p<0.001, ***p<0.0005, ****p<0.0001.

We next determined whether the enhanced expression of CAT1 in the host cell was a crucial determinant for *Toxoplasma* infection. Using CRISPR/Cas9, we knocked out the *CAT1* gene in a population of MEF cells, leading to sharply lowered levels of *CAT1* mRNA and protein **(Fig 5A and S8A Fig)**. Confirming the specificity of the CAT1-targeted deletion, levels of *SLC7A2* and *SLC7A3* mRNAs remained similar to wild-type (WT) MEF cells during the course of *Toxoplasma* infection in the CAT1 knockout cells **(S8B and S8C Fig)**.

**Fig 6:**
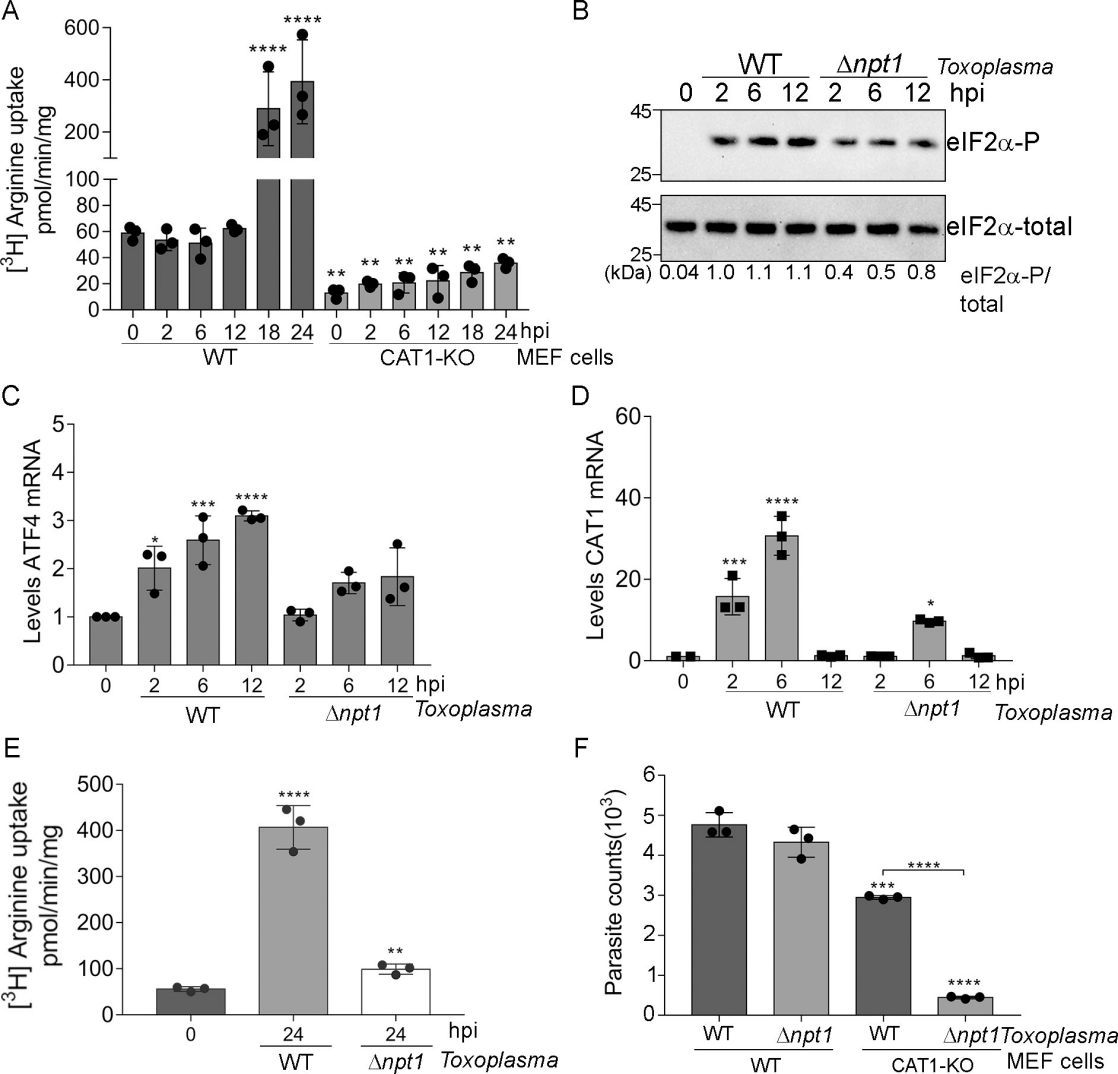
Arginine uptake in host cells during *Toxoplasma* infection. **(A)** Uptake of radiolabeled arginine was measured during *Toxoplasma* infection in WT or CAT1-KO MEF cells. Note the measurements were for an 8 minute addition of [^3^H]-arginine from the medium into wild-type Values of infected cells were normalized to mock-infected cells (±SD, n = 3) **p<0.001, ****p<0.0001. **(B)** Wild-type MEF cells were infected with *Toxoplasma* lacking arginine transporter TgNPT1 (*Δnpt1*) or the parental strain (designated WT) using a MOI of 3:1. At the indicated hpi, infected cells were harvested and the levels of eIF2α-P and total eIF2α were measured by immunoblot (n = 3). The values below eIF2α-P/eIF2α total indicate band intensities. Wild-type MEF cells were infected with parental (WT *Toxoplasma* or *Δnpt1* in RPMI medium and the levels of **(C)** *ATF4* and **(D)** *CAT1* mRNAs were measured by RT-qPCR. Values of infected cells were normalized to mock-infected cells (±SD, n = 3) *p<0.005, ***p<0.0005 and ****p<0.0001. **(E)** Uptake of radiolabeled arginine was measured 24 hpi with either parental (WT) or *Δnpt1* in MEF cells cultured in RPMI medium. Values of infected cells were normalized to mock-infected cells (±SD, n = 3) *p<0.005 and ****p<0.0001. **(F)** Parental (WT) or *Δnpt1* parasites were used in infect WT or CAT1-KO MEF cells cultured in RPMI. At 30 hpi, genomic DNA was isolated and the number of parasites was determined by a qPCR assay (±SD, n = 3) **p<0.001, ***p<0.0005, ****p<0.0001.

Loss of CAT1 led to sharply reduced arginine levels in the MEF cells, which were exacerbated upon *Toxoplasma* infection **(Fig 5B)**. Consequently, parasite replication was significantly compromised in CAT1-depleted host cells **(Fig 5C, S6F, S6G and S8D Figs)**. Supplementing the CAT1-deficient host cells with additional arginine partially rescues parasite replication, suggesting that arginine uptake can take place at least in part by alternative transporters **(Fig 6A and S8F Fig)**. These results support the model that increased expression of *CAT1* by the ISR ensures that host cells can provide sufficient arginine for replication of *Toxoplasma*.

*Toxoplasma* encodes four different protein kinases that phosphorylate the parasite eIF2α (TgIF2α) and confer translational control [17]. Each of these *Toxoplasma* TgIF2α kinases serve in stress adaptation, with two nonessential GCN2-related variants designated TgIF2K-C and TgIF2K-D functioning during nutrient deprivation in the parasite [18,19]. We reasoned that if *Toxoplasma* infection of CAT1-depleted host cells led to arginine depletion in the host and subsequently the parasite, then the parasite GCN2-related protein kinases would be critical for *Toxoplasma* replication. In agreement with our earlier studies that TgIF2K-C and TgIF2K-D are not essential for parasite replication in HFFs grown in normal culture conditions [18,19], deletion of either of these GCN2-related protein kinases in *Toxoplasma* had no effect on parasite replication in wild-type MEF cells expressing CAT1 **(Fig 5C)**. However, *Toxoplasma* lacking either TgIF2K-C or TgIF2K-D showed reduced replication in CAT1-depleted host cells compared to WT MEF cells **(Fig 5C)**. These results indicate that eIF2α-P plays a pivotal role in nutrient sensing and adaptation in both parasite and host cells.

### Arginine uptake in host cells during *Toxoplasma* infection

We next determined whether activation of the ISR and the ensuing enhancement of CAT1 alters arginine uptake by the host cell during *Toxoplasma* infection. We monitored the transport of [^3^H]-arginine from the medium into WT and CAT1-knockout MEF cells over a time course of *Toxoplasma* infection. Note that the radiolabelled arginine was applied to the cultured cells for 8 minutes to measure the efficiency of arginine transport at the indicated hpi. WT MEF cells showed modest arginine transport prior to infection, which increased >50-fold by 18 hours of infection **(Fig 6A)**. By comparison, cells with diminished levels of CAT1 showed low arginine transport, even at later time points of infection. These findings support the critical role of CAT1 for arginine uptake in MEF cells infected with *Toxoplasma*.

Next, we addressed the contribution of the selective arginine transporter in *Toxoplasma*, TgNPT1, for salvaging arginine from the host cell [3]. We reasoned that if the parasites take up less arginine from the host cells, there would be diminished induction of the host ISR during the course of parasite infection. Consistent with this model, we found that MEF cells infected with Δ*npt1* parasites for up to 12 hpi showed 50% lowered induction of eIF2α-P compared to those cells infected with WT parasites in RPMI medium (Δ*npt1* parasites must be cultured in RPMI rather than DMEM, as RPMI has higher arginine concentration [3]) **(Fig 6B).** Furthermore, there was a delay and a reduced induction of *ATF4* and *CAT1* mRNA in the host cells infected with Δ*npt1* parasites **(Fig 6C and 6D)**. We also confirmed that lowered CAT1 expression in host cells infected with Δ*npt1* parasites led to sharply lowered arginine transport into infected host cells **(Fig 6E)**. Collectively, these findings indicate that the host ISR is activated by parasite-dependent depletion of host arginine, which culminates in the host cell enhancing CAT1-dependent transport of the amino acid. The interplay between the arginine transporters of the parasite (TgNPT1) and host cell (CAT1) is important for parasite replication. Whereas deletion of host *CAT1* partially lowered parasite counts, the combined loss of host *CAT1* and the parasite *NPT1* sharply ablated parasite replication **(Fig 6F)**.

### Discussion

**Fig 7:**
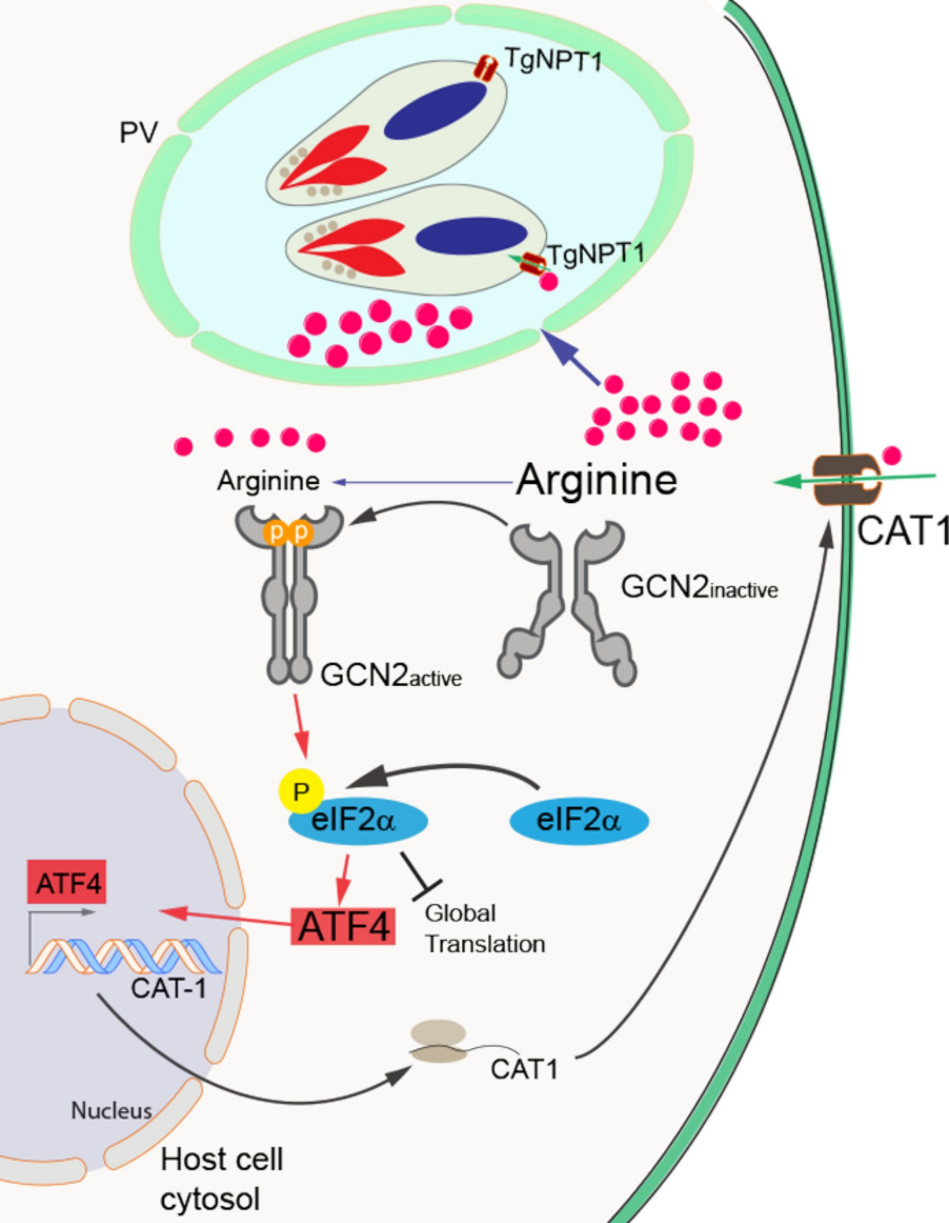
Host GCN2 and the downstream ISR effectors ATF4 and CAT1 help secure arginine for replication of *Toxoplasma*. *Toxoplasma* (represented as two tachyzoites residing within a parasitophorous vacuole (PV) inside a host cell) requires arginine and other nutrients from the host cell for replication. During *Toxoplasma* infection, the ensuing depletion of arginine in the host cell activates GCN2 to phosphorylate eIF2α, which lowers global translation accompanied by induced preferential translation of *ATF4*. ATF4 directly enhances the transcription of *CAT1*, encoding a cationic amino acid transporter that facilitates arginine import into host cells, thus securing a continued supply of this essential nutrient for the intracellular parasites. *Toxoplasma* NPT1 facilitates arginine transport from host cells to the parasite, although it is noted that there may be other mechanisms that have yet to be elucidated for parasites to obtain arginine. Deletion of *GCN2*, *ATF4*, or *CAT1* in the host cells inhibits *Toxoplasma* replication.

*Toxoplasma* and other obligate intracellular parasites satisfy their resource needs by appropriating essential nutrients from their host cells. However, by doing so the parasites can quickly deplete available nutrients in the host cell, which would jeopardize parasite survival and replication. This study describes an intricate balance between *Toxoplasma* and host that ensures that a continual supply of nutrients is available for parasite replication. As illustrated in the model represented in Fig 7, *Toxoplasma* is auxotrophic for certain amino acids, and upon infection can rapidly deplete arginine levels in host cells via its arginine transporter TgNPT1 **(Figs 3A and 6)**. We note that it was recently reported that *Toxoplasma* may also acquire arginine through the ingestion of host proteins [20]. Deprivation of amino acids can induce the host ISR, featuring GCN2-mediated phosphorylation of eIF2α, which enhances expression of ATF4 **(Fig 1)**. ATF4 directly induces transcriptional expression of genes involved in the uptake and synthesis of amino acids, including the cationic amino transporter CAT1. Furthermore, *CAT1* translation was reported to be enhanced by eIF2α-P during amino acid limitations [21]. The ensuing increase in CAT1 leads host cells to take up more arginine, unwittingly securing a constant stream of this critical amino acid for the parasites growing within **(Fig 3)**. The finding that addition of both arginine and tryptophan further lowered eIF2α-P during *Toxoplasma* infection compared to arginine alone **(Fig 3B–3D)** suggests that regulatory interplay between the host ISR and parasite is applicable to other amino acids for which the parasite is an auxotroph.

Activation of a cascade of host ISR factors function to ensure that *Toxoplasma* is supplied with necessary nutrients required for replication. Loss of host GCN2, or its downstream targets ATF4 and CAT1 **(Figs 2J and 5C and S6G and S8E Figs)**, sharply reduces *Toxoplasma* replication. It is noteworthy that while GCN2 is the first responder in the host ISR during parasite infection, a second eIF2α kinase PERK is activated later during *Toxoplasma* infection, suggesting that an ER stress is experienced by host cells as parasite numbers expand **(Fig 2)**. The ER stress in host cells appears to involve calcium release from this organelle and may be a consequence of parasitophorous vacuole enlargement and/or its association with the host ER [22]. The parasitophorous vacuole is critical for parasite nutrient acquisition from the host cell, and our findings show that the GCN2/ATF4/CAT1 pathway in the host ISR facilitates a steady supply of amino acids to the parasite.

Analogous to the host ISR, *Toxoplasma* also senses nutrient depletion via its GCN2-related kinases TgIF2K-C and TgIF2K-D. Deletion of either of these TgIF2α kinases had no effect on parasite replication in WT MEFs cultured in DMEM; however, loss of either TgIF2K-C or TgIF2K-D significantly lowered parasite replication in MEF cells depleted for CAT1 **(Fig 5C)**. Interestingly, TgIF2K-C appeared to be more critical than TgIF2K-D when cultivated in CAT1-depleted host cells. The regulatory mechanisms for these TgIF2α kinases have yet to be resolved, but we have previously shown that TgIF2K-C responds to amino acid deprivation experienced by intracellular parasites [19]. In contrast, TgIF2K-D appears to be necessary to maintain the fitness of extracellular parasites [18]. Parasite amino acid transporters, such as TgNPT1, are also crucial for salvaging nutrients from the host **(Fig 6)**, and it would be of interest to determine whether the TgIF2Ks contribute to the expression and function of these transporters upon nutrient stresses. Considered together, our findings highlight the intricate balance between parasite and host, with each possessing complex nutrient responsive systems involving translational control that function together to optimize parasite survival. The complexity of these pathways provide for potential therapeutic intervention to subvert the ability of intracellular parasites such as *Toxoplasma* to thrive.

Our findings bolster a growing body of literature describing how pathogens manipulate host cell translation during their intracellular stages. Viruses, bacteria, and other intracellular pathogens have been shown to regulate translation through the ISR or mTOR signaling pathway using effector proteins or by creating a nutrient imbalance [23]. Our study is the first to demonstrate that apicomplexan parasites hijack components of host translational control to ensure nutrient acquisition by the parasite. In this case, it does not appear that a parasite effector protein is involved, but rather the host ISR is triggered in response to the parasite appropriating host nutrients.

## Materials and methods

### Host cell and parasite culture

Wild-type MEF (mouse embryonic fibroblast) and *GCN2*^-/-^, *PERK*^-/-^, *GCN2*^-/-^/*PERK*^-/-^, *PKR*^-/-^, and *GCN2*^-/-^/*PERK*^-/-^/*PKR*^-/-^ counterparts [10], along with HFF (human foreskin fibroblast) cells and J774A.1 macrophages were maintained in Dulbecco’s modification of Eagle’s medium (DMEM) supplemented with 10% heat-inactivated fetal bovine serum (FBS) (Gibco/Invitrogen) and penicillin/streptomycin at 37°C with 5% CO_2_. The lack of host eIF2 kinase(s) had no detectable effect on ability of *Toxoplasma* to invade the mutant host cells **(S4A Fig)**. The *ATF4*^*-/-*^ MEF cells were cultured in DMEM that was supplemented with nonessential amino acids, and 55 μM β-mercaptoethanol (Sigma-Aldrich) as described [10]; the media was adjusted to the standard pH 7.2 and filtered before use. Prior to infection, host cells were split to a density of 2x10^5^ cells/well and cultured overnight. Infection media consisted of DMEM supplemented with 1% FBS; mock infection showed that these conditions resulted in negligible eIF2α-P **(S1F Fig)**. Type I RH strain *Toxoplasma* parasites were used at a multiplicity of infection (MOI) of 3:1. The WT (TATi/Δ*ku80*) and Δ*npt1* parasites (a gift from Dr. Giel van Dooren, Australian National University) were cultured in RPMI medium as described [3]. RPMI medium contains additional nutrients, including arginine, which helps to overcome *Toxoplasma* growth defects associated with loss of *NPT1* observed for infected cells cultured in DMEM [3]. Infected cells were harvested at the indicated times in RIPA buffer supplemented with cOmplete and EDTA-free Protease Inhibitor Cocktail (Roche) following of protein quantification by Bradford Reagent (Sigma-Aldrich) then the proteins were separated by SDS-PAGE.

### Puromycin incorporation

To determine the levels of protein synthesis during *Toxoplasma* infection, MEF cells were infected with *Toxoplasma* and at the indicated hpi, the infected cells were then incubated with 10 μg/mL puromycin (Sigma-Aldrich) for 15 min. Infected cells were harvesting, and total protein lysates were analyzed by immunoblot analyses using the anti-puromycin antibody (EMD Millipore). Protein synthesis was quantified by densitometry using Image J and normalized by eIF2α. The Infected cells supplemented with arginine were incubated with puromycin as a control **(S1 Fig)**.

### Arginine measurement assay

Relative arginine levels were measured in host cells using a colorimetric arginase activity assay kit according to manufacturer’s instructions (Abcam ab180877). The infected cells were washed with PBS and scraped from the plates. Following centrifugation, the pellet was resuspended in assay buffer with 0.01% of Triton X-100 to disrupt the host cell membrane. A second centrifugation step was performed to remove host cell debris and parasites, with the supernatant containing the host cell cytosol fraction used to perform the assay shown in S4B Fig. Fidelity of the purification was assayed by immunoblot for parasite surface antigen SAG1 and host eIF2α **(S4B Fig)**. Supernatant samples were diluted 1:10 and 40 μL was used for the assay. After 10 min of incubation with the arginase enzyme, absorbance was measured at 570 nm in kinetic mode for 30 min in a BioTek microtiter plate reader. The values were compared with an arginine standard curve.

### Calcium measurement assay

Calcium was measured by the Calcium Assay kit (colorimetric, (Abcam ab102505). For this measurement, 2x10^6^ cells were infected with *Toxoplasma* for up to 24 h and then harvested in PBS containing 0.04% of digitonin on ice. Following centrifugation, the resulting supernatant represented the cytosolic fraction **(S4C Fig)**, which was diluted 1:50 in dH_2_O and adjusted to 50 μL/well following the manufacturer’s instructions. Absorbance was measured in a BioTek microplate reader at OD575 nm. For controls, uninfected cells were treated with 1 μM tunicamycin for 2 h, 1 μM thapsigargin for 2 h, or 1 μM A23187 for 5 min. Alternately, calcium levels in the uninfected and infected MEF cells were measured with cell permeant Fluo-4, AM (Thermo Fisher Scientific, F14201) according to the manufacturer’s instructions **(S4D Fig)**.

### Generation of knockdown cells

Deletion of the *CAT1* (*SLC7A1*) gene in a population of MEF cells was performed using CRISPR/Cas9, generating CAT1-KO cells. Briefly, four different sgRNAs (g1-ATGGGCTGCAAAAACCTGCTCGG, g2-CCAGGACTTACCGATGATGTAGG, g3-CACAAACGTGAAATACGGTGAGG, g4-CATCATGAGCGTGAGAGCGGCGG) were prepared using the EnGen sgRNA Synthesis Kit (New England BioLabs). The individual sgRNAs were associated with EnGen Cas9 NLS protein (New England BioLabs), which were then transfected into MEF cells using the 4D-Nucleofector System (Lonza) in combination with the P4 Primary Cell 4D-Nucleofector X Kit. As a negative control, EnGen sgRNA Control Oligo (CATCCTCGGCACCGTCACCC) was associated with Cas9 NLS and transfected into MEF cells. Targeted cell lines transfected with one of the sgRNAs, or a combination of all four, were validated by RT-qPCR using specific primers **(S8A Fig)** and by immunoblot using antibody that specifically recognizes CAT1 (Abcam ab37588).

### Quantification of parasites

A PCR-based assay was used to determine the number of parasites in host cells as previously described [24]. Briefly, host cells were infected with *Toxoplasma* at a MOI 3:1; at 2 hpi, the infection medium was replaced with fresh DMEM. At 30 hpi, genomic DNA was isolated and measured by quantitative PCR using primers to a parasite-specific gene region designated B1 [25]. We note that it is technically difficult to enumerate tachyzoites growing inside of MEF cells as they are not as easily visualized as in larger HFF cells. Therefore, to independently verify changes in parasite growth between WT and CAT1-KO MEF cells, equal numbers of parasites were allowed to infect the MEF host cells for 24 hours. Infected MEFs were then scraped and the material passed through a syringe; equal portions of the lysate preparations were then used to infect HFF cells. Five days post-infection, parasite viability was assessed using a standard plaque assay.

### Immunoblot analyses

Equal amounts of protein lysates were separated by SDS-PAGE, and immunoblot analyses were carried out for three independent experiments using horseradish peroxidase–tagged secondary antibody. Primary antibodies used for immunoblots included total eIF2α (Cell Signaling Technology, #9722), eIF2α-P (Cell Signaling Technology, #9721), custom affinity-purified ATF4 antibody [26], CAT1 (Abcam, ab37588), GAPDH (Abcam, ab9485), puromycin (EDM Millipore, #17H1), and p70 S6 Kinase (49D7) rabbit mAb (Cell Signaling Technology #2708). Blots were incubated with Pierce ECL Western Blotting Substrate prior to imaging on FluorChem M- Multiplex fluorescence (Protein Simple).

### Measurement of mRNA levels

2x10^5^ MEF cells were plated in 6-well plates and allowed to adhere overnight. Cells were infected with tachyzoites for 2 h, washed in PBS, then cultured in DMEM for the indicated times. RNA was isolated from the infected cells using TRIzol LS Reagent (Invitrogen) and cDNA was generated using Omniscript (Qiagen). RT-qPCR was carried out using primers specific to the indicated gene transcript (Table 1), in combination with SYBR Green Real-Time PCR Master Mixes (Invitrogen) and StepOnePlus Real System. Relative levels of transcripts were calculated with the *ΔΔCt* method using genes encoding GAPDH and β-actin as internal controls. The relative levels of the target mRNAs from the mock-infected samples were adjusted to 1 and served as the basal control value. Each experiment was performed three times, each with three technical replicates.

**Table 1:** List of primers used in this study.

Primer name	Forward/ Reverse	Sequence (5’-3’)	Reference PMID
**ATF4**	F	GCCGGTTTAAGTTGTGTGCT	23761072
**ATF4**	R	CTGGATTCGAGGAATGTGCT	23761072
**Xbp1-total**	F	AAGAACACGCTTGGGAATGG	21917591
**Xbp1-total**	R	ACTCCCCTTGGCCTCCAC	21917591
**Xbp1-splicing**	F	GAGTCCGCAGCAGGTG	21917591
**Xbp1-splicing**	R	CTCTGGGAGTTCCTCCAGACT	21917591
**CAT1**	F	CTTGGACCAGTGCAAATGACG	16670299
**CAT1**	R	TGATCCTGAGGCATGAGTGCA	16670299
**Slc7a2**	F	GTGAAGAGGTTCGGAATCCACA	16670299
**Slc7a2**	R	CGTTAAAGCTGCAGA	16670299
**Slc7a3**	F	GGCTCCCTCTGTGCACTTTCTA	16670299
**Slc7a3**	R	TAGCAAGGACACGGAACAGGA	16670299
β‐actin	F	TGTTACCAACTGGGACGACA	23761072
***β-actin***	R	GGGGTGTTGAAGGTCTCAAA	23761072
GAPDH	F	TCACCACCATGGAGAAGGC	27620138
***GAPDH***	R	GCTAAGCAGTTGGTGGTGCA	27620138

### Immunofluorescence assay

MEF cells were infected with *Toxoplasma* for 24h and then were fixed with 4% paraformaldehyde for 20 minutes and blocked with PBS supplemented with 2% BSA. Cells were permeabilized in blocking buffer containing 0.1% Triton X-100 for 30 min then incubated with rabbit anti-CAT1 (Abcam) and mouse anti-SAG1 (Invitrogen) for 1 hour. Alternatively, the cells were incubated with anti-CAT1 without permeabilization, followed by incubation with anti-SAG1. Secondary goat anti-rabbit Alexa-fluor 488 and anti-mouse Alexa-594 (Invitrogen) was applied for 1 hour followed by Vectashield mounting media. DAPI was used as a co-stain to visualize host and parasite nuclei (Vector Labs). Images were acquired with Leica inverted DMI6000B microscope with 63x oil immersion objective.

### Arginine uptake assay

Radiolabeled arginine uptake assays were based on previously published methods [27–29]. Briefly, MEF cells were infected with *Toxoplasma* for the designated time points. Infected cells were then incubated with 0.5 μCi [^3^H]-arginine (Perkin Elmer) in HEPES buffer with 5.6 mM D-glucose at pH 7.4, 24°C. After 8 min, uptake of radiolabelled arginine was thwarted by incubating the cells with 50 mM L-arginine. Arginine uptake was terminated by rapidly washing the cells with ice-cold HEPES buffer following lysis, with 1 ml of 0.5% SDS in 0.5 N NaOH. 700 μl of the lysate was mixed with 5.2 mL of scintillation buffer and read for 1 min in the Packard 1600TR Liquid Scintillation Counter. The remaining sample aliquot was used to determine protein concentration.

### Quantification and statistical analysis

Quantitative data are presented as the mean and standard deviation from biological triplicates. Statistical significance was determined using One-way ANOVA with Tukey's post hoc test and multiple t-test in Prism (version 7) software (GraphPad Software, Inc., La Jolla, CA). The number of biological replicates (n) and p values are indicated in the legend of each figure. For immunoblot analyses, the reported images are representative of at least three independent experiments.

## Supporting information

S1 Fig Puromycin incorporation in infected cells. **(A)** Total protein synthesis was measured in mock-infected or **(B)** MEF cells infected with *Toxoplasma* for the indicated time. Translation was measured by incubating cells with puromycin for 15 min, followed by lysate preparation and immunoblot with puromycin-specific antibodies (±SD, n = 3). **(C)** Total protein synthesis during *Toxoplasma* infection with or without arginine supplementation in the media. Below each puromycin immunoblot panel is an immunoblot measurement of total eIF2α protein.
(TIF)

S2 Fig *Toxoplasma* infection triggers eIF2α phosphorylation in other host cells. **(A)** HFF cells and **(B)** J774.1 macrophages were infected with *Toxoplasma* and levels of eIF2α-P and total eIF2α were measured by immunoblot at indicated times. *ATF4* mRNA levels in HFF cells **(C)** and J774.1 macrophages **(D)** infected with *Toxoplasma* for the indicated times were measured by RT-qPCR; values were normalized to mock-infected cells (±SD, n = 3) ****p<0.0001. **(E)** HEK293T cells were infected with *Toxoplasma* and at the indicated hpi, and infected cells were harvested and the levels of eIF2α-P and total eIF2α were measured by immunoblot analyses. **(F)** MEF cells were infected or mock-infected and harvested at the indicated time points to assay the levels of eIF2α-P and total eIF2α by immunoblot.
(TIF)

S3 Fig Effect of *Toxoplasma* infection on *ATF4* mRNA in MEF cells deficient for individual or combinations of eIF2 kinases. *ATF4* mRNA levels were measured by RT-qPCR in MEF cells lacking **(A)** GCN2 **(B)** PERK **(C)** GCN2 and PERK **(D)** PKR, or **(E)** the combination of GCN2, PERK and PKR. Values were normalized to mock-infected cells (±SD, n = 3) **p<0.001, ***p<0.0005 and ****p<0.0001.
(TIF)

S4 Fig Cellular fractionation controls and calcium levels in infected cells. **(A)** A dual-staining assay was used to determine the percent of parasites that had invaded WT MEF cells or those deleted individually or in combination for the indicated eIF2α kinases. **(B)** MEF cells were lysed in assay buffer solution supplemented with 0.01% Triton X-100, then cytosol and pellet were separated by centrifugation. SAG1 (*Toxoplasma gondii* P30) and cytosolic host eIF2α were measured by immunoblot to verify purity of the fractions **(C)** MEF cells were lysed in a solution containing 0.04% digitonin for 10 min on ice, and the cytosol and pellet were separated by centrifugation. The ER-resident chaperone protein BiP (GRP78/HSPA5) and cytosolic eIF2α were assayed by immunoblot to verify purity of the fractions. **(D)** WT MEF cells infected with *Toxoplasma* for the indicated times were incubated with Fluo-4-AM (±SD, n = 3) *p<0.01, **p<0.001, ***p<0.0005, ****p<0.0001. For controls, uninfected cells were treated with 1 μM of the SERCA inhibitor thapsigargin (TG) for 1 h or 1 μM of the calcium ionophore A23187 for 5 min.
(TIF)

S5 Fig Levels of *ATF4* mRNA during amino acid supplementation in MEF cells infected with *Toxoplasma*. *ATF4* mRNA levels were measured by RT-qPCR in *Toxoplasma*-infected MEF cells supplemented with 10-fold the amount of the indicated amino acid present in DMEM. Levels of *ATF4* mRNA were normalized to mock-infected cells (±SD, n = 3) **p<0.001, ***p<0.0005.
(TIF)

S6 Fig Impact of *Toxoplasma* infection on CAT1 protein expression in WT and mutant MEF cells. **(A)** IFA for CAT1 protein (green) and *Toxoplasma* (red) in WT and CAT1-KO MEF cells infected with *Toxoplasma* in presence or absence of permeabilization as indicated (60X magnification). DAPI (blue) was used as a co-stain to highlight host (large) and parasite nuclei (small). Note that CAT1 levels are increased throughout the host cells during infection, not only in the portions of the host cells where the parasites are located. Levels of CAT1 protein were measured by immunoblot at the indicated hpi of parasite in **(B)** *GCN2*^-/-^, **(C)** *ATF4*^-/-^, or **(D)** *PERK*^-/-^ MEF cells **(E)** Levels of CAT1 protein measured in WT MEF cells treated with ISRIB. As a normalization control, GAPDH protein levels were measured in the same lysate preparations (n = 3). Quantitation represents the band intensity of CAT1 protein normalized for GAPDH. **(F)** The diagram outlines the experimental design for a parasite plaque assay that measures viability of parasites derived from infected WT or CAT1-KO MEF cells. The MEF cells were infected with equal numbers of parasites for 24 hours, followed by scrape/syringe lysis. Equal portions of the lysates were then used to infect HFF cells. **(G)** Five days post-infection, host cell lysis was determined by plaque assay.
(TIF)

S7 Fig Levels of *SLC7A2* and *SLC7A3* mRNAs do not significantly change during infection. **(A)** *SLC7A2* and **(B)** *SLC7A3* mRNAs were measured by RT-qPCR in WT MEF cells infected with *Toxoplasma* for the indicated times. The bar graph represents relative mRNA levels normalized to zero (uninfected) with error bars representing standard deviation (n = 3).
(TIF)

S8 Fig Depletion of *CAT1* in MEF cells by CRISPR/Cas9. **(A)** MEF cells were transfected with one of four different sgRNA-CAT1, a mixture of all four, or a sgRNA control. RNA was isolated from each of the transfected cell populations and the levels of *CAT1* mRNA were measured by RT-qPCR. The bar graph represents relative *CAT1* mRNA levels normalized to the zero (uninfected) time point (±SD, n = 3), ***p<0.0005. Levels of **(B)** *SLC7A2* and **(C)** *SLC7A3* mRNAs were measured in WT or CAT1-KO cells infected with *Toxoplasma* for the indicated times (±SD, n = 3). **(D)** MEF cells depleted for CAT1 by CRISPR/Cas9 (CAT1-KO) were infected with *Toxoplasma*; at the indicated times, genomic DNA was isolated and parasite counts were determined by qPCR (±SD, n = 3), ***p<0.0005. **(E)** MEF cells lacking ATF4 were infected with *Toxoplasma*. At 30 hpi, genomic DNA was extracted to measure the number of replicating parasites in the host cells using qPCR. Data were analyzed with multiple t-test (±SD, n = 3) ****p<0.0001. **(F)** WT and CAT1-KO MEF cells were infected with *Toxoplasma* in presence or absence of arginine supplementation. At 30 hpi, genomic DNA was extracted to measure the number of replicating parasites in the host cells using qPCR. Data were analyzed with multiple t-test (±SD, n = 3) ****p<0.0001, ***p<0.0005, and *p<0.01.
(TIF)
