# Supplementary figures and images for: Regulation of arginine transport by GCN2 eIF2 kinase is important for replication of the intracellular parasite Toxoplasma gondii
Source: PLoS Pathog. 2019 Jun 13;15(6):e1007746. doi: 10.1371/journal.ppat.1007746 (PMC6564765; doi:10.1371/journal.ppat.1007746)

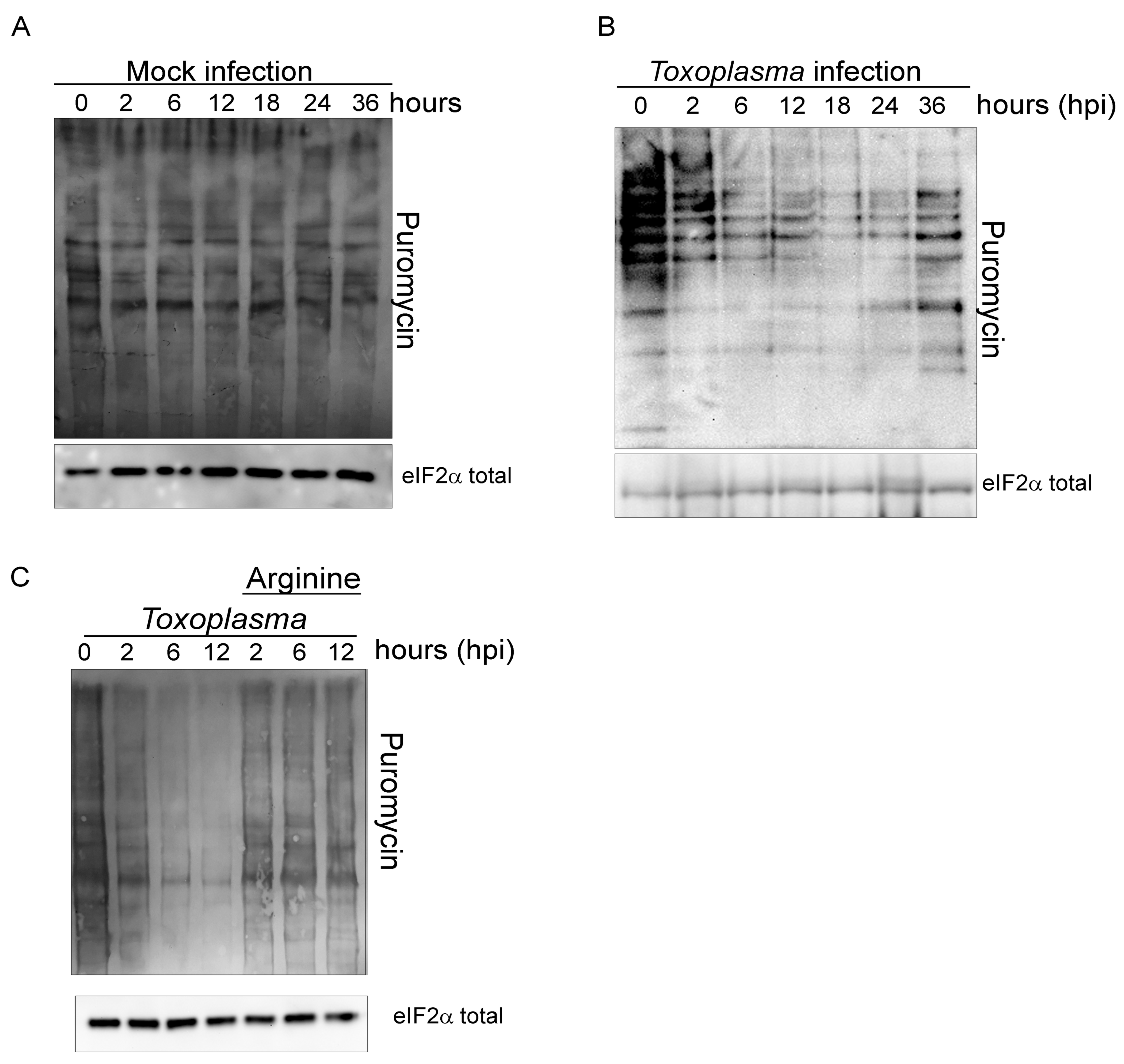

Supplement: S1 Fig — (A) Total protein synthesis was measured in mock-infected or (B) MEF cells infected with Toxoplasma for the indicated time. Translation was measured by incubating cells with puromycin for 15 min, followed by lysate preparation and immunoblot with puromycin-specific antibodies (±SD, n = 3). (C) Total protein synthesis during Toxoplasma infection with or without arginine supplementation in the media. Below each puromycin immunoblot panel is an immunoblot measurement of total eIF2α protein. (TIF) [file ppat.1007746.s001.tif]

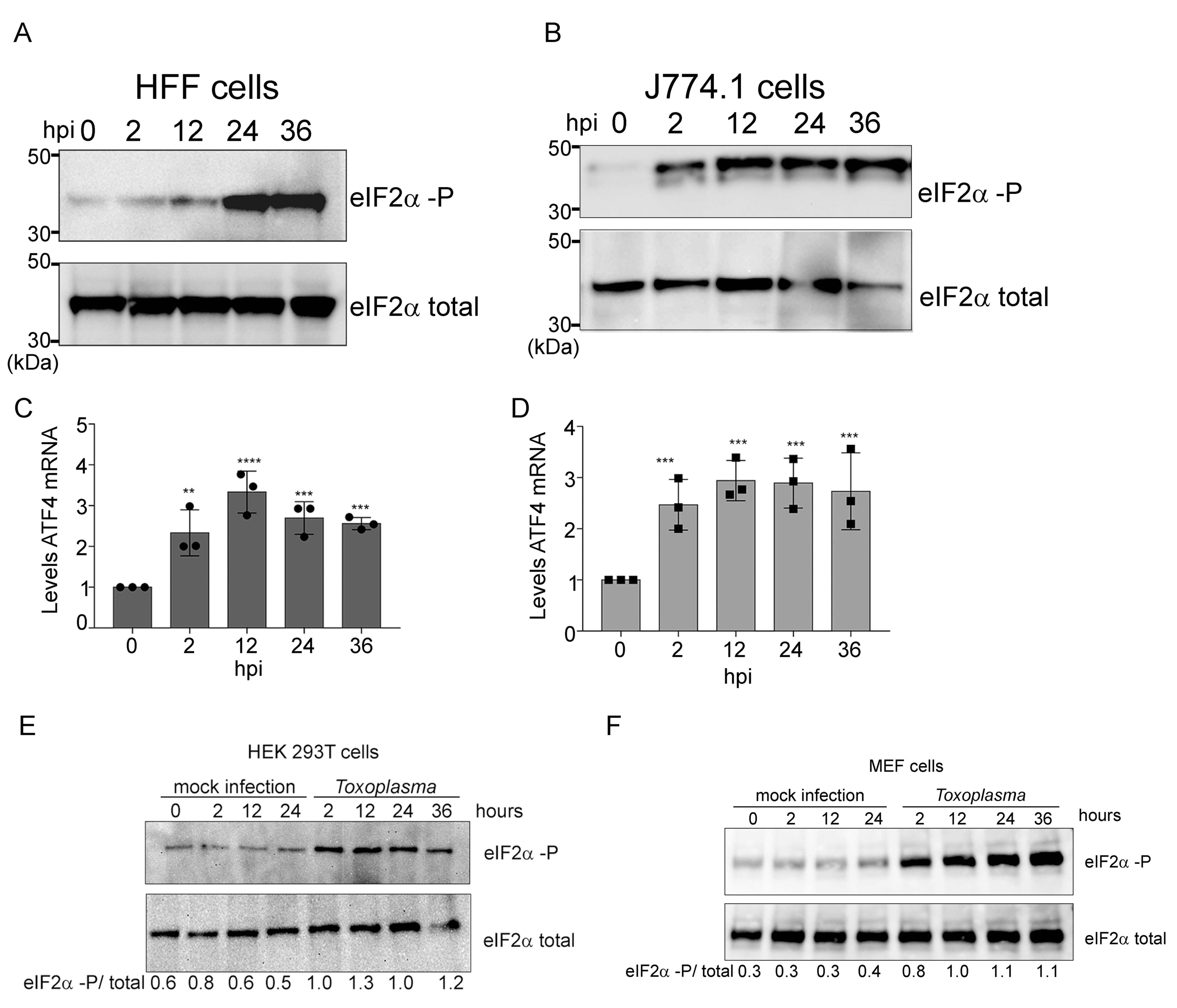

Supplement: S2 Fig — (A) HFF cells and (B) J774.1 macrophages were infected with Toxoplasma and levels of eIF2α-P and total eIF2α were measured by immunoblot at indicated times. ATF4 mRNA levels in HFF cells (C) and J774.1 macrophages (D) infected with Toxoplasma for the indicated times were measured by RT-qPCR; values were normalized to mock-infected cells (±SD, n = 3) ****p<0.0001. (E) HEK293T cells were infected with Toxoplasma and at the indicated hpi, and infected cells were harvested and the levels of eIF2α-P and total eIF2α were measured by immunoblot analyses. (F) MEF cells were infected or mock-infected and harvested at the indicated time points to assay the levels of eIF2α-P and total eIF2α by immunoblot. (TIF) [file ppat.1007746.s002.tif]

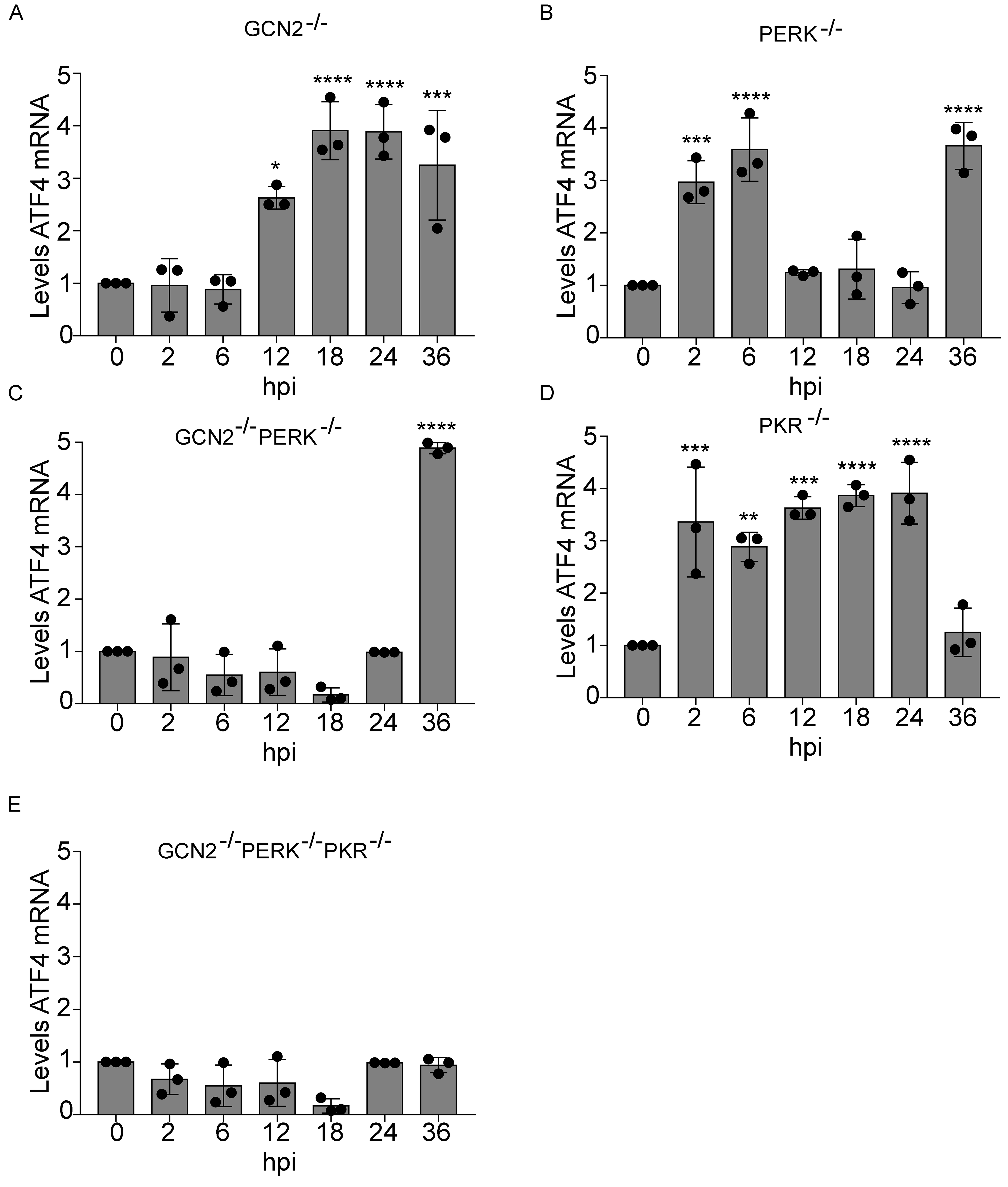

Supplement: S3 Fig — ATF4 mRNA levels were measured by RT-qPCR in MEF cells lacking (A) GCN2 (B) PERK (C) GCN2 and PERK (D) PKR, or (E) the combination of GCN2, PERK and PKR. Values were normalized to mock-infected cells (±SD, n = 3) **p<0.001, ***p<0.0005 and ****p<0.0001. (TIF) [file ppat.1007746.s003.tif]

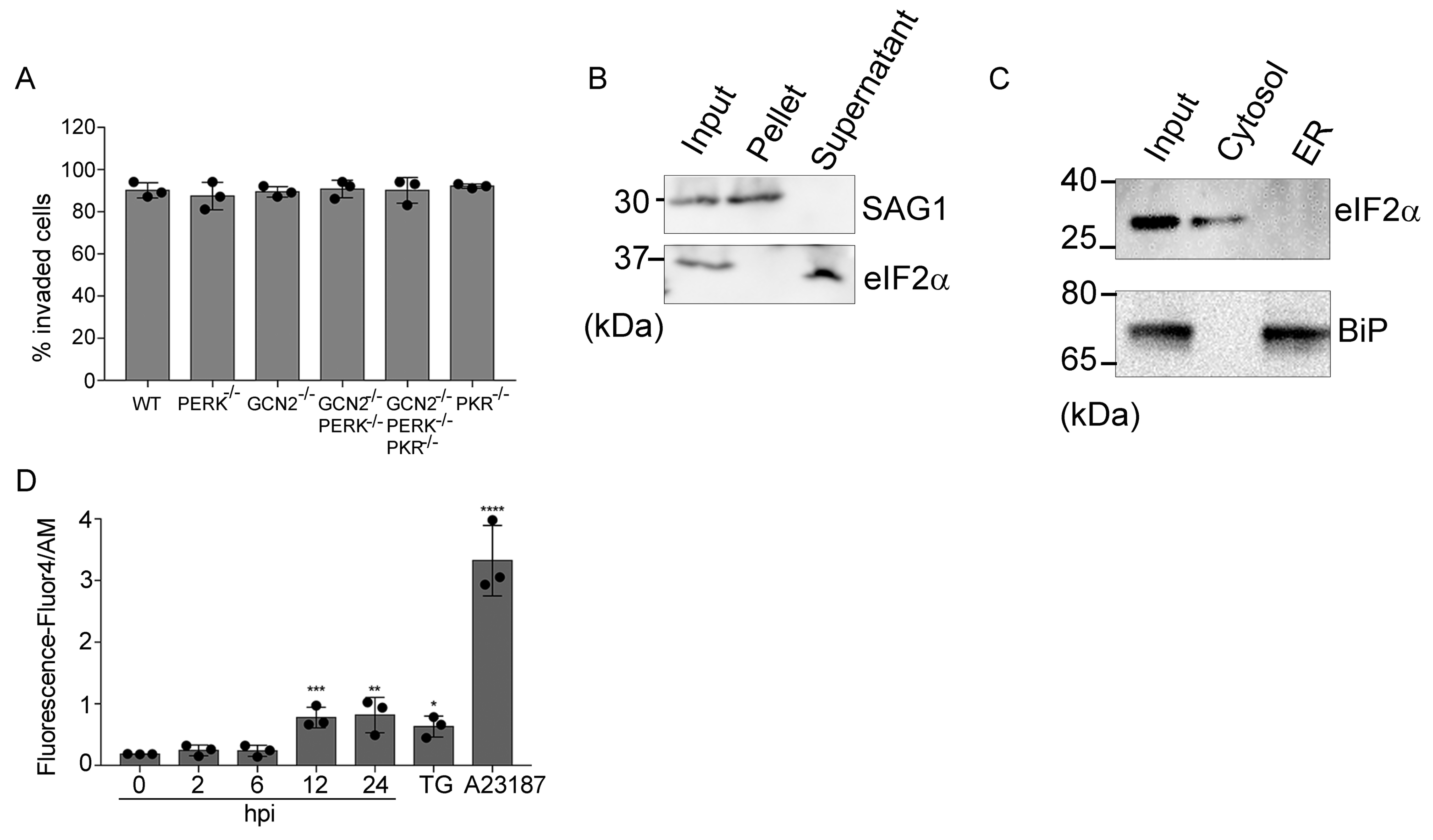

Supplement: S4 Fig — (A) A dual-staining assay was used to determine the percent of parasites that had invaded WT MEF cells or those deleted individually or in combination for the indicated eIF2α kinases. (B) MEF cells were lysed in assay buffer solution supplemented with 0.01% Triton X-100, then cytosol and pellet were separated by centrifugation. SAG1 (Toxoplasma gondii P30) and cytosolic host eIF2α were measured by immunoblot to verify purity of the fractions (C) MEF cells were lysed in a solution containing 0.04% digitonin for 10 min on ice, and the cytosol and pellet were separated by centrifugation. The ER-resident chaperone protein BiP (GRP78/HSPA5) and cytosolic eIF2α were assayed by immunoblot to verify purity of the fractions. (D) WT MEF cells infected with Toxoplasma for the indicated times were incubated with Fluo-4-AM (±SD, n = 3) *p<0.01, **p<0.001, ***p<0.0005, ****p<0.0001. For controls, uninfected cells were treated with 1 μM of the SERCA inhibitor thapsigargin (TG) for 1 h or 1 μM of the calcium ionophore A23187 for 5 min. (TIF) [file ppat.1007746.s004.tif]

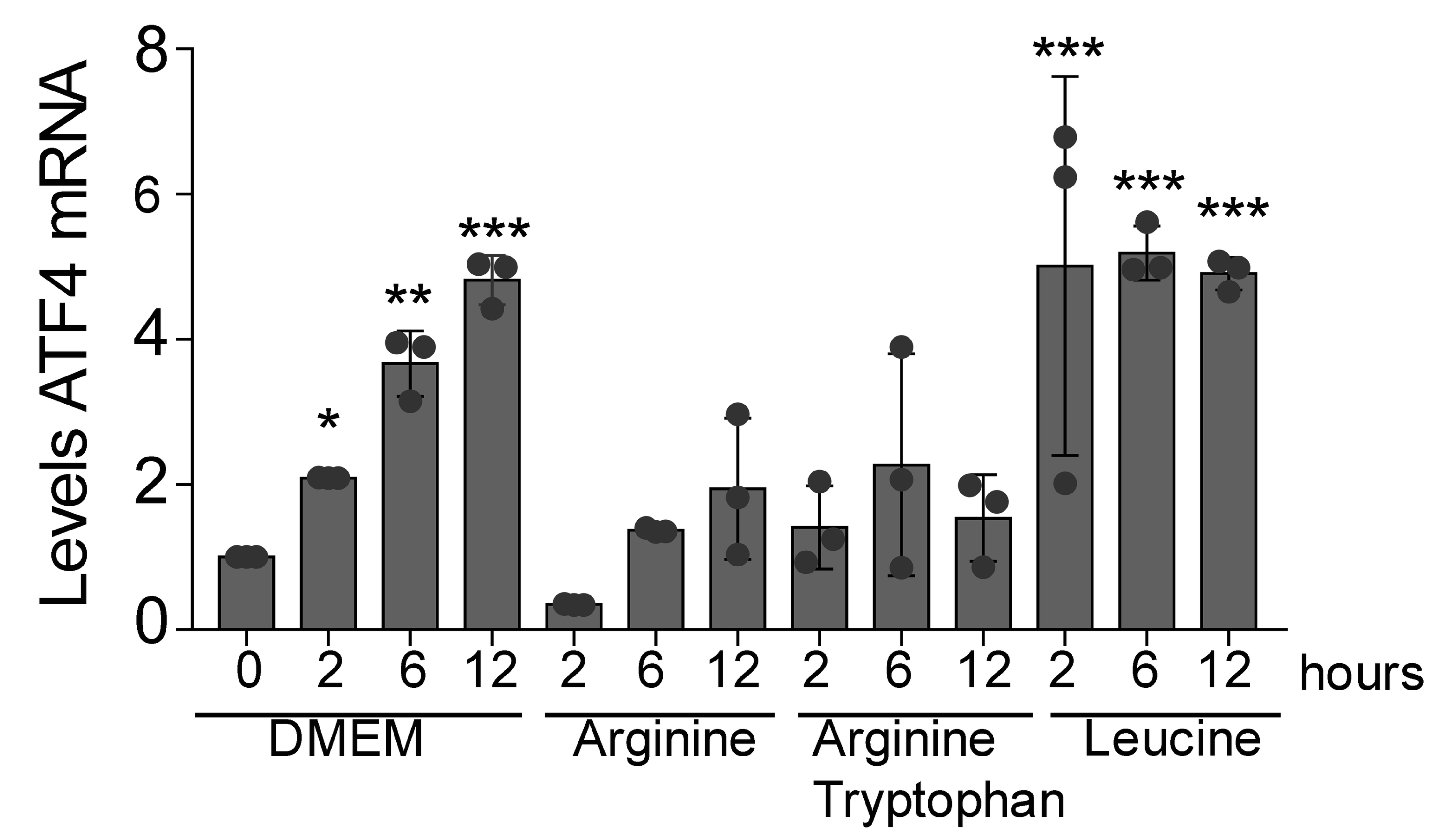

Supplement: S5 Fig — ATF4 mRNA levels were measured by RT-qPCR in Toxoplasma-infected MEF cells supplemented with 10-fold the amount of the indicated amino acid present in DMEM. Levels of ATF4 mRNA were normalized to mock-infected cells (±SD, n = 3) **p<0.001, ***p<0.0005. (TIF) [file ppat.1007746.s005.tif]

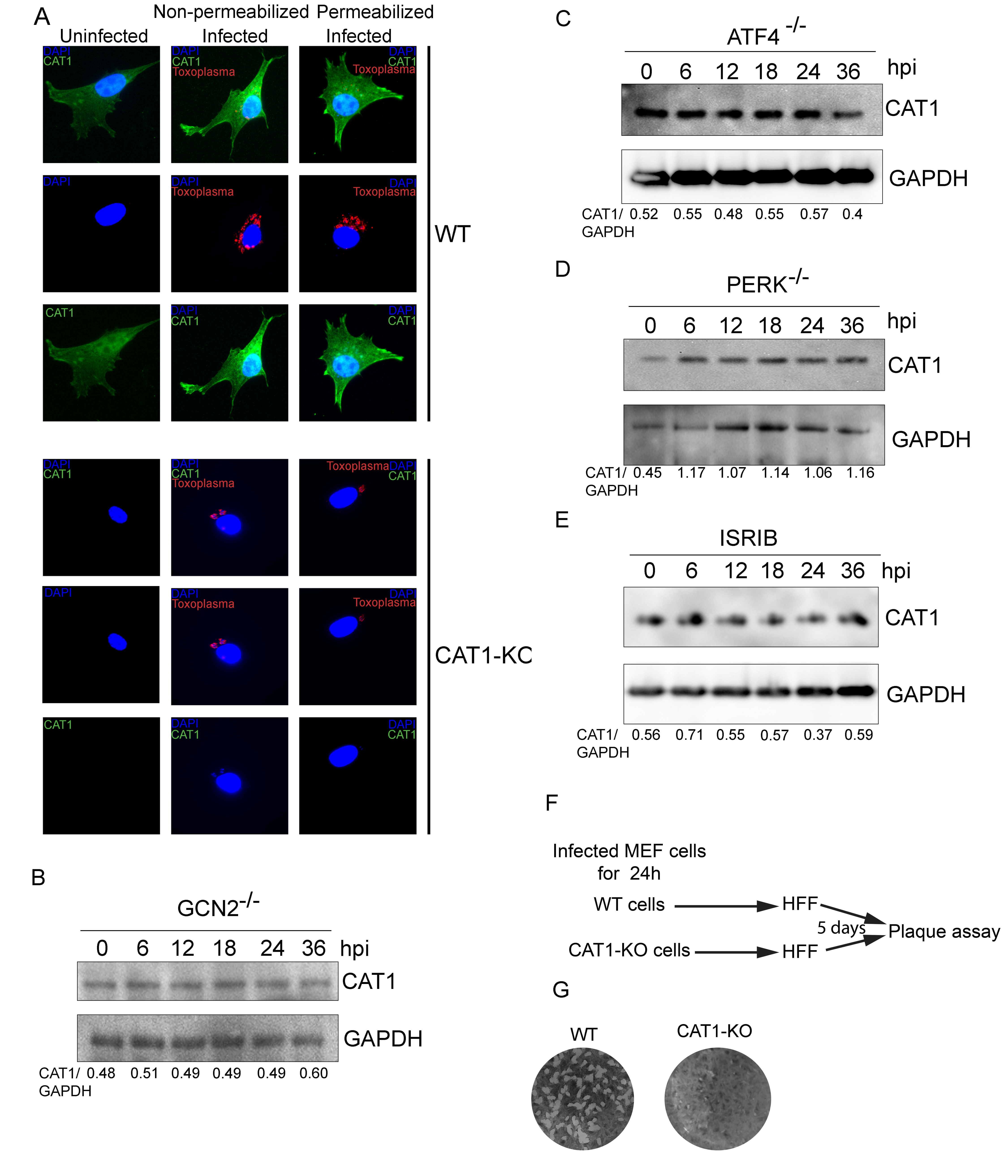

Supplement: S6 Fig — (A) IFA for CAT1 protein (green) and Toxoplasma (red) in WT and CAT1-KO MEF cells infected with Toxoplasma in presence or absence of permeabilization as indicated (60X magnification). DAPI (blue) was used as a co-stain to highlight host (large) and parasite nuclei (small). Note that CAT1 levels are increased throughout the host cells during infection, not only in the portions of the host cells where the parasites are located. Levels of CAT1 protein were measured by immunoblot at the indicated hpi of parasite in (B) GCN2-/-, (C) ATF4-/-, or (D) PERK-/- MEF cells (E) Levels of CAT1 protein measured in WT MEF cells treated with ISRIB. As a normalization control, GAPDH protein levels were measured in the same lysate preparations (n = 3). Quantitation represents the band intensity of CAT1 protein normalized for GAPDH. (F) The diagram outlines the experimental design for a parasite plaque assay that measures viability of parasites derived from infected WT or CAT1-KO MEF cells. The MEF cells were infected with equal numbers of parasites for 24 hours, followed by scrape/syringe lysis. Equal portions of the lysates were then used to infect HFF cells. (G) Five days post-infection, host cell lysis was determined by plaque assay. (TIF) [file ppat.1007746.s006.tif]

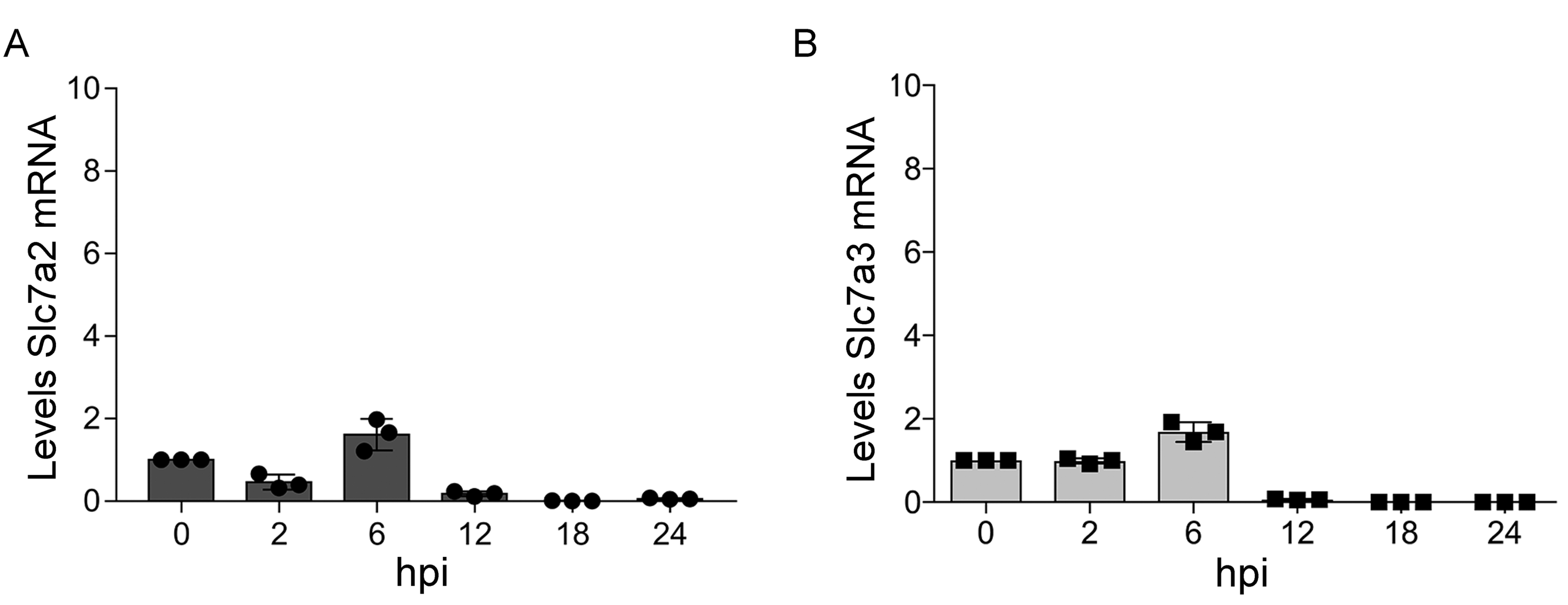

Supplement: S7 Fig — (A) SLC7A2 and (B) SLC7A3 mRNAs were measured by RT-qPCR in WT MEF cells infected with Toxoplasma for the indicated times. The bar graph represents relative mRNA levels normalized to zero (uninfected) with error bars representing standard deviation (n = 3). (TIF) [file ppat.1007746.s007.tif]

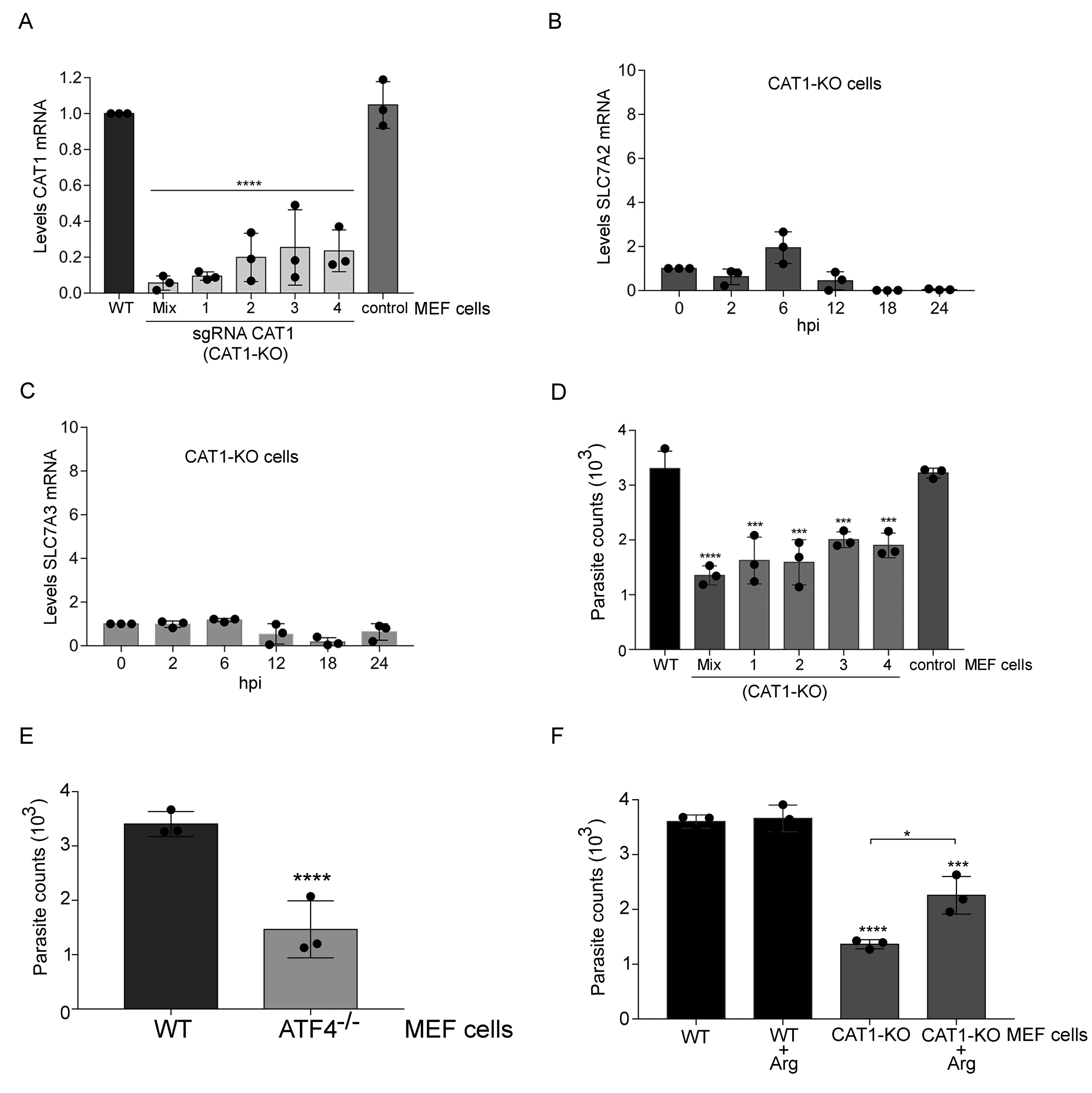

Supplement: S8 Fig — (A) MEF cells were transfected with one of four different sgRNA-CAT1, a mixture of all four, or a sgRNA control. RNA was isolated from each of the transfected cell populations and the levels of CAT1 mRNA were measured by RT-qPCR. The bar graph represents relative CAT1 mRNA levels normalized to the zero (uninfected) time point (±SD, n = 3), ***p<0.0005. Levels of (B) SLC7A2 and (C) SLC7A3 mRNAs were measured in WT or CAT1-KO cells infected with Toxoplasma for the indicated times (±SD, n = 3). (D) MEF cells depleted for CAT1 by CRISPR/Cas9 (CAT1-KO) were infected with Toxoplasma; at the indicated times, genomic DNA was isolated and parasite counts were determined by qPCR (±SD, n = 3), ***p<0.0005. (E) MEF cells lacking ATF4 were infected with Toxoplasma. At 30 hpi, genomic DNA was extracted to measure the number of replicating parasites in the host cells using qPCR. Data were analyzed with multiple t-test (±SD, n = 3) ****p<0.0001. (F) WT and CAT1-KO MEF cells were infected with Toxoplasma in presence or absence of arginine supplementation. At 30 hpi, genomic DNA was extracted to measure the number of replicating parasites in the host cells using qPCR. Data were analyzed with multiple t-test (±SD, n = 3) ****p<0.0001, ***p<0.0005, and *p<0.01. (TIF) [file ppat.1007746.s008.tif]
